# Visualizing K48 Ubiquitination during Presynaptic Formation By Ubiquitination-Induced Fluorescence Complementation (UiFC)

**DOI:** 10.3389/fnmol.2016.00043

**Published:** 2016-06-10

**Authors:** Maria J. Pinto, Joana R. Pedro, Rui O. Costa, Ramiro D. Almeida

**Affiliations:** ^1^Center for Neuroscience and Cell Biology (CNC), University of CoimbraCoimbra, Portugal; ^2^PhD Programme in Experimental Biology and Biomedicine (PDBEB), Center for Neuroscience and Cell Biology, University of CoimbraCoimbra, Portugal; ^3^School of Allied Health Technologies, Polytechnic Institute of Porto (ESTSP-IPP)Vila Nova de Gaia, Portugal; ^4^Institute for Interdisciplinary Research, University of CoimbraCoimbra, Portugal

**Keywords:** ubiquitination, presynaptic terminal, presynaptic differentiation, axon development, lysine 48 polyubiquitin

## Abstract

In recent years, signaling through ubiquitin has been shown to be of great importance for normal brain development. Indeed, fluctuations in ubiquitin levels and spontaneous mutations in (de)ubiquitination enzymes greatly perturb synapse formation and neuronal transmission. In the brain, expression of lysine (K) 48-linked ubiquitin chains is higher at a developmental stage coincident with synaptogenesis. Nevertheless, no studies have so far delved into the involvement of this type of polyubiquitin chains in synapse formation. We have recently proposed a role for polyubiquitinated conjugates as triggering signals for presynaptic assembly. Herein, we aimed at characterizing the axonal distribution of K48 polyubiquitin and its dynamics throughout the course of presynaptic formation. To accomplish so, we used an ubiquitination-induced fluorescence complementation (UiFC) strategy for the visualization of K48 polyubiquitin in live hippocampal neurons. We first validated its use in neurons by analyzing changing levels of polyubiquitin. UiFC signal is diffusely distributed with distinct aggregates in somas, dendrites and axons, which perfectly colocalize with staining for a K48-specific antibody. Axonal UiFC aggregates are relatively stable and new aggregates are formed as an axon grows. Approximately 65% of UiFC aggregates colocalize with synaptic vesicle clusters and they preferentially appear in the axonal domains of axo-somatodendritic synapses when compared to isolated axons. We then evaluated axonal accumulation of K48 ubiquitinated signals in bead-induced synapses. We observed rapid accumulation of UiFC signal and endogenous K48 ubiquitin at the sites of newly formed presynapses. Lastly, we show by means of a microfluidic platform, for the isolation of axons, that presynaptic clustering on beads is dependent on E1-mediated ubiquitination at the axonal level. Altogether, these results indicate that enrichment of K48 polyubiquitin at the site of nascent presynaptic terminals is an important axon-intrinsic event for presynaptic differentiation.

## Introduction

Neurons are highly complex and polarized cells with a remarkable network of functionally active processes that extend outwards the cell body. Within the brain, each neuron’s axon establishes thousands of synaptic contacts with neighboring or fairly distantly located neurons. Differentiation of presynaptic terminals occurs early in development (Steward and Falk, 1991; Bury and Sabo, 2010) and it comprises recruitment and coordinated clustering of presynaptic material that can be found along the axon in the form of cell body-derived mobile units (Jin and Garner, 2008; Pinto and Almeida, 2016). Specificity to this phenomenon is conferred by cues derived from the postsynaptic partner that by activating axonal receptors trigger presynaptic assembly (Johnson-Venkatesh and Umemori, 2010; Siddiqui and Craig, 2011). Despite the huge number of proteins known to be implicated in presynaptic differentiation, this is a highly rapid event that occurs in a time-scale of minutes to few hours (Friedman et al., 2000; Bresler et al., 2004). Furthermore, axons are extremely long and presynaptic terminals are to be formed in remote sites. On top of this, each presynaptic site is an individual micro-domain, meaning that changes in one do not necessarily affect adjacent segments. In light of these circumstances, axons are believed to rely on intra-axonal mechanisms to support and sustain their prompt response to cues, subsequently leading to a site-specific clustering of presynaptic components. Indeed, there are predefined sites along the axon shaft in which *en passant* presynaptic terminals selectively form (Krueger et al., 2003; Sabo et al., 2006), thus establishing the importance of intrinsic axonal mechanisms.

Ubiquitin is a highly conserved small protein that is covalently attached to other proteins in the form of a single monomer, monoubiquitination, or as a chain of ubiquitins, polyubiquitination (Komander and Rape, 2012). All seven internal lysines in ubiquitin can serve as attachment sites for other ubiquitins, and so, different chain types can be formed, which differently alter properties of the target protein and are involved in a multitude of cellular processes (Komander and Rape, 2012; Sadowski et al., 2012). Of particular relevance is its role as a tag for proteasome-mediated degradation mainly through lysine 48 and 11-linked polyubiquitin chains, in the so-called ubiquitin-proteasome system (UPS; Kulathu and Komander, 2012; Kleiger and Mayor, 2014).

Although very much less explored, signaling through ubiquitin is also likely to play a role in presynapse development. The ataxia mice ax^J^, with a loss-of-function mutation in the proteasome-associated deubiquitinating enzyme Usp14 and concomitant decreased synaptic levels of monomeric and conjugated ubiquitin, display severe malformation of the neuromuscular junction and impaired presynaptic function (Wilson et al., 2002; Chen et al., 2009). These defects are rescued by restoration of ubiquitin levels (Chen et al., 2011). Contrariwise, transgenic mice overexpressing ubiquitin also display impaired formation of presynapses (Hallengren et al., 2013), thus reinforcing that tightly balanced ubiquitin levels are crucial for proper synaptic development. Furthermore, similar presynaptic defects are also observed in mice carrying mutations in the E3 ubiquitin ligases HERC1 (Bachiller et al., 2015) and PHR (Burgess et al., 2004; Saiga et al., 2009). Interestingly, the *Drosophila* and *C. elegans* homologs of PHR have been shown to function locally in modulating the triggering cascades that guide presynaptic differentiation (Liao et al., 2004; Nakata et al., 2005; Collins et al., 2006). Altogether, these observations point to a fundamental role for ubiquitination in the events launching presynaptic assembly.

Notwithstanding, the mechanistic role of ubiquitin in vertebrate presynaptic formation is still unclear. We have made significant advances in the field by demonstrating that proteasome-related polyubiquitin signals trigger presynaptic assembly (Pinto et al., 2016), which is in line with the higher expression of lysine 48 ubiquitin chains at the peak of synapse formation (Chen et al., 2011) and the high number of embryonic ubiquitinated proteins involved in synaptogenesis (Franco et al., 2011). In the study reported here, we exploited the ubiquitination-induced fluorescence complementation (UiFC) assay (Chen et al., 2013) to look closely to K48 ubiquitination along axons and its relation to sites of presynaptic clustering. In contrast to previous ubiquitin-based fluorescence complementation approaches, which allow for detection of substrate-specific ubiquitination (Fang and Kerppola, 2004; Kerppola, 2006), UiFC detects endogenous conjugation of K48 ubiquitin chains due to favored binding of UiFC’s ubiquitin-interacting motifs and reconstitution of fused Venus fragments (Chen et al., 2013). Using UiFC in neuronal cultures, we show that axonal aggregates of enhanced K48 polyubiquitination are mostly stable and preferably located at axo-somatodendritic regions. Moreover, the majority colocalizes with presynaptic clusters. We further observed that axonal localized enrichment of K48 polyubiquitin occurs rapidly upon contact with a synaptic partner. Lastly, we demonstrate that clustering of presynaptic material requires ubiquitination at the axon level. Overall, we propose that site-specific enrichment of K48 polyubiquitinated conjugates supports presynaptic formation and this effect is dependent on axonal E1-mediated ubiquitination.

## Materials and Methods

### Primary Neuronal Cultures

Animals were maintained at the animal house of the Center for Neuroscience and Cell Biology (University of Coimbra, Portugal) approved by the Portuguese National Authority for Animal Health (DGAV). Primary cultures of rat hippocampal neurons were prepared from E17 Wistar rat embryos, as previously described (Baptista et al., 2010; Baeza et al., 2012) with minor changes. Hippocampi were dissected and dissociated in 0.045% trypsin/0.01% v/v deoxyribonuclease in HBSS for 15 min at 37°C. Then washed once in plating media (supplemented with 0.026 MEM NaHCO3, 0.025 M glucose, 1 mM sodium pyruvate and 10% FBS), mechanically dissociated and cell density determined. Cells were diluted in plating medium and plated in poly-D-lysine (PDL)-coated surfaces. Plating medium was replaced by culture medium (neurobasal medium supplemented with 2% B27, 25 μM glutamate, 0.5 mM glutamine and 1:400 penicillin-streptomycin) 2–4 h after plating. Cells were maintained in a humidified incubator with 5% CO_2_/95% air at 37°C. The mitotic inhibitor 5-FDU was added at days *in vitro* (DIV) 3/4. Experiments were performed at DIV 7/8.

### Microfluidic Devices

Microfluidic devices were prepared by assembling a molded poly-dimethylsiloxane (PDMS) chamber onto a glass coverslip (Taylor et al., 2005; Pinto et al., 2016). The molds for the PDMS devices used in this study were fabricated by Noo Li Jeon (School of Mechanical and Aerospace Engineering, Seoul National University, Seoul, Korea). PDMS was prepared from the Sylgard 184 Silicone elastomer kit (Dow Corning), poured onto the microfluidic molds and cured for 4–6 h. PDMS devices were assembled on top of glass coverslips (Marienfeld) coated with PDL and laminin.

### Neuron Transfection

F(syn)WRBN-VGluT1mCherry, a vector for expression of a fusion version of VGluT1 to mCherry controlled by the synapsin promoter, was kindly offered by Prof. Etienne Herzog (Interdisciplinary Institute for Neuroscience, Bordeaux, France; Herzog et al., 2011). The constructs for ubiquitination-induced fluorescence complementation (UiFC), pcDNA3-UiFC-C (UiFC-C) and pcDNA3-UiFC-N (UiFC-N), were kindly offered by Prof. Shengyun Fang (Department of Biochemistry and Molecular Biology, University of Maryland, Baltimore, Maryland, MD, USA; Chen et al., 2013). Also, pcDNA3.1-GFP and pcDNA3.1-mCherry were used for the expression of GFP and mCherry, respectively. Deoxyribonucleic acid (DNA) was recombinantly expressed in primary hippocampal neurons using calcium phosphate transfection at DIV 6/7 (Almeida et al., 2005). Expression was allowed to occur for 16–20 h.

### PDL-Coated Beads and Drugs

PDL-coated beads were prepared at the experiment day. 4.5 μm-diameter aliphatic amine latex beads (Life Technologies) were incubated with PDL for 30 min at 37°C, washed twice in sterile mQH_2_O and diluted in culture medium or HEPES-buffered solution imaging medium (119 mM NaCl, 5 mM KCl, 2 mM CaCl_2_, 2 mM MgCl_2_, 30 mM glucose, 10 mM HEPES, pH 7.4) for experiments requiring fixation or live imaging, respectively. Beads were added to cultures for the indicated periods of time and incubated at 37°C. Drug treatment [IU1 (75 μM, Tocris Bioscience), MG132 (1μM, Calbiochem), PR619 or Ziram (1μM, Sigma Aldrich)] was performed in conditioned medium (either culture or imaging medium) by diluting the drug from a 1000×-concentrated stock in DMSO. Equal amounts of DMSO were added to the control condition. Ziram was always prepared fresh before experiment.

### Immunocytochemistry and Image Acquisition

Immunostaining of cultured neurons was performed similarly to previously described (Baptista et al., 2013). Fixation in 4% paraformaldehyde (in PBS with 4% sucrose) for 10 min was followed by washes in PBS. Cells were then permeabilized in PBS with 0.25% Triton X-100 for 5 min, washed once and blocking performed with 3% BSA for at least 30 min. These steps were performed at room temperature. Incubation with primary antibodies in 3% BSA was performed either overnight at 4°C or for 2 h at 37°C followed by three washes in PBS. Secondary antibodies were incubated for 1 h at room temperature in 3% BSA and washed (twice with PBS with 0.1% Triton X-100 and once with PBS). The following primary antibodies were used: Bassoon (1:400; #ADI-VAM-PS003; Enzo Life Sciences), GFP (1:1000, #598, MBL), K48 polyubiquitin (Apu2; 1:500; #05-1307, Millipore), MAP2 (1:5000; #AB5543; Chemicon), tau (1:1000; #AB75714; Abcam) and ubiquitin (1:200; #Z0458; Dako, Denmark). Alexa-conjugated secondary antibodies (405, 488, 568 and 647) were used (1:1000; Life Technologies).

Imaging was performed either in a Zeiss Observer Z.1 microscope equipped with a Plan-Apochromat 20× air objective (0.8 NA) or a Plan-NeoFluar 63× oil objective (1.4 NA), an AxioCam HRm camera and Zen Blue 2011 software or in a spinning disk confocal imaging system (CSU-X1M, Yokogawa) configured to a Zeiss Axio Observer Z1 microscope with a LCI Plan-NeoFluar 63× water or glycerol objective (1.3 NA) coupled to an EM-CCD Evolve Delta camera and Zen Black 2012 Software. All conditions within an experiment were processed simultaneously and imaging settings (exposure time and laser power) were conserved. The spinning disk system was used for fixed preparations with beads and for obtaining *XY* reconstructions of microfluidic devices (with both somal and axonal compartments). For the experiment in which we were interested in determining the density of axonal UiFC and VGluT1mCherry puncta at sites with or without somatodendritic contact, *XY* reconstructed images were obtained with the 63× objective in the spinning disk.

### Live-Imaging

Experiments involving live cells were all performed in a spinning disk confocal imaging system (CSU-X1M, Yokogawa) configured to a Zeiss Axio Observer Z1 microscope with a LCI Plan-NeoFluar 63× water or glycerol objective (1.3 NA) coupled to an EM-CCD Evolve Delta camera and Zen Black 2012 Software. All experiments were done in imaging medium at 37°C in a humidified atmosphere to avoid medium evaporation. Correction for focal drift was accomplished by the definite focus feature of Zen Software. *z*-stacks encompassing the sample of interest were acquired at each time frame. Images were obtained every 1, 5 or 10 min depending on the experiment. Drug treatment or addition of beads was preceded by acquisition of three frames.

### Quantitative Analysis

Except for analysis of kymographs, quantification was performed using ImageJ Software. All images were converted to 8-bit for quantification purposes. To quantify the number of UiFC aggregates, K48 aggregates and VGluT1mCherry puncta, dendritic and/or axonal markers were used to select branches to quantify and their length determined. Particle analysis was applied to UiFC, K48 ubiquitin and VGluT1mCherry images after appropriately thresholded to quantify number of puncta in each neuronal segment of known length. Due to differences in the expression level of the reporters, threshold to identify puncta was differently adjusted in each image so that distinct puncta would be considered whilst discarding diffuse signal along the axon shaft. In order to determine the density of axonal UiFC and VGluT1mCherry puncta, at sites with or without contact, somas were identified in the brightfield image and their ROIs used to determine whether an axonal domain was in contact with somas (axo-somatic) or isolated. Identification of axo-somatodendritic and axo-dendritic domains was carried out by staining for MAP2 and creating ROIs encompassing MAP2-positive structures. The length of “axo-somatodendritic” and “isolated” axons was determined and used to quantify the density of puncta within each domain. To quantify the number of UiFC-VGluT1mCherry clusters, the presence or absence of UiFC aggregates within VGluT1mCherry puncta ROIs was determined. For quantifying signal intensity on beads, raw intensity values of the signal of interest (UiFC, VGluT1mCherry and stained Bassoon, K48 ubiquitin and tau) within ROIs encompassing beads was quantified from *z*-projections (sum of all slices) of original *z*-stacks. Signal at equal-sized off-bead ROIs adjacent to each bead was also quantified. When comparing changes in Bassoon, K48 ubiquitin and tau on beads at different time-points and upon ziram treatment, background signal was subtracted and the ratio of signal intensity between on-bead and the correspondent off-bead calculated.

For quantification of changes in time-lapse series, *z*-stacks were sum projected and aligned with the StackReg plugin. The brightfield image was used to locate somas or beads in contact with axons and ROIs created. Fluorescence intensity within ROIs was measured in each frame of the time-lapse series and normalized to the frame preceding drug treatment or addition of beads (0 min). Kymograph analysis was performed in ICY Software. Kymographs were extracted from axonal segments from a 1 h time-lapse (frames every 1 min). The kymograph tracking tool was used to trace the path of UiFC aggregates on kymographs. For each aggregate, the net run length and mean instant speed were quantified. Puncta with mean instant speed values greater than 0.05 μm/min and net displacements greater than twice their width (limit set to 0.08 μm because UiFC aggregates’ average diameter is 0.04 μm) were considered as mobile. Per each axonal segment, the number of mobile and stable puncta was calculated.

All images were processed and prepared for presentation using Photoshop and Illustrator (Adobe).

### Statistical Analysis

Unless otherwise indicated, results are presented as averaged values ± SEM. Graphs and statistical analysis were performed in Graph Pad Prism 5 Software. Statistical differences were examined by non-parametric tests (the test used for each experiment is indicated in the figure legends). Values of *p* < 0.05 were considered statistically significant (*p < 0.05; **p < 0.01 and ***p < 0.001).

## Results

### An Ubiquitin-Induced Fluorescence Complementation (UiFC) Approach to Monitor K48 Ubiquitination in Axons

Bimolecular fluorescence complementation has been extensively used in neurons (Feinberg et al., 2008; Unoki et al., 2012; Ramaker et al., 2013; Das et al., 2015; Macpherson et al., 2015) to achieve spatiotemporal resolution in the unmasking of cellular and synaptic aspects. Herein, we pioneered the use of an ubiquitin-based fluorescence complementation approach in neuronal cells to look deep into ubiquitination phenomena in the developing axon. The recently developed UiFC assay allows for live monitoring of K48 polyubiquitination and consists of two constructs, UiFC-C and UiFC-N, each bearing ubiquitin interacting motifs fused to either the N- or C-terminal non-fluorescent fragments of Venus (Chen et al., 2013). Upon polyubiquitination, preferably on lysine 48, interaction of ubiquitin interacting motifs with growing chains reconstitutes Venus fluorescence, whose appearance can be detected within 10 min (Chen et al., 2013).

**Figure 1:**
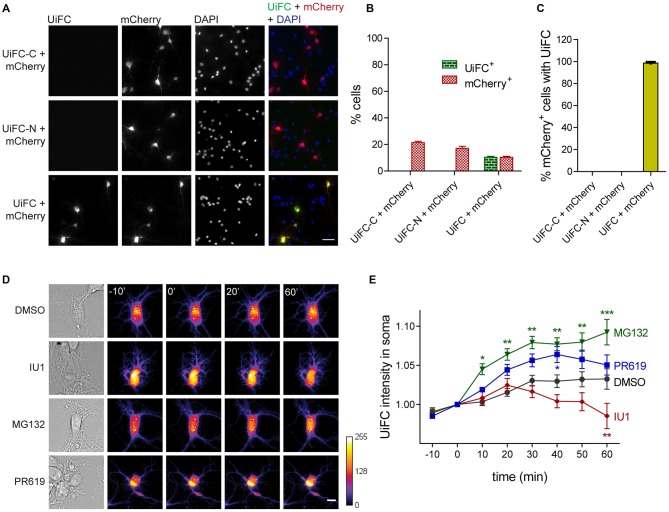
**Expression and validation of ubiquitination-induced fluorescence complementation (UiFC) in hippocampal neurons. (A)** Expression of UiFC in hippocampal neurons. The two constructs for the UiFC assay, UiFC-C and UiFC-N, were transfected in hippocampal neurons by calcium phosphate transfection either independently or in combination. To control for transfection efficacy, mCherry was also expressed in each condition. The total amount of deoxyribonucleic acid (DNA) added as well as the image acquisition settings (gain and exposure time) were kept constant between conditions. Reconstitution of Venus fluorescence in neurons was observed when UiFC-C and UiFC-N were co-transfected, but not when each plasmid was expressed alone. Scale bar represents 50 μm. **(B)** Percentage of cells expressing UiFC and mCherry. **(C)** Percentage of mCherry-expressing neurons showing UiFC signal. **(B,C)** A total of 40 fields of view (FOV) were analyzed per condition from two independent experiments. **(D)** Time-lapse imaging of changes in UiFC signal on neuronal somas. Neuronal cultures expressing UiFC (firelut) were treated with DMSO, IU1 (75 μM, proteasome activator), MG132 (1 μM, proteasome inhibitor) and PR619 (1 μM, deubiquitinases inhibitor) and images were taken every 10 min. Expression of UiFC in neurons was sensitive to changes in ubiquitin-proteasome system (UPS) function and subsequent ubiquitination levels. Scale bar represents 10 μm. **(E)** Quantitative changes in UiFC signal per soma area. Results are normalized to the frame preceding treatment (0 min) and shown as Mean ± SEM. Statistical significance by 2-way ANOVA (***p < 0.001, **p < 0.01 and *p < 0.05 when compared to DMSO at each time-point). A total of 18 (DMSO), 27 (IU1), 35 (MG132) and 18 (PR619) somas were analyzed from three independent experiments.

We first asked if ubiquitination-induced Venus reconstitution was observed in primary hippocampal neurons upon expression of UiFC plasmids. To accomplish so, cells were singly or doubly transfected with UiFC-C and/or UiFC-N. To control for transfection efficacy, co-transfection with mCherry was performed. Transfection of each UiFC plasmid alone did not yield any Venus fluorescent signal despite the presence of mCherry-expressing cells (Figures 1A,B). However, when UiFC-C and UiFC-N were co-transfected approximately 10% of cells displayed fluorescence for UiFC (Figures 1A,B), thus demonstrating that the signal specifically reveals sites of Venus reconstitution. Importantly, approximately 100% of mCherry-expressing cells exhibit Venus signal when both UiFC constructs were expressed (Figure 1C), clearly showing that lack of Venus signal upon single UiFC-C or UiFC-N expression is not due to absence of transfected cells.

We then assessed UiFC ability to faithfully recapitulate changes in cellular ubiquitination levels. To accomplish so, we performed time-lapse imaging in cultured neurons expressing UiFC upon treatment with specific UPS drugs that alter the lifetime of ubiquitinated conjugates (Figure 1D). We used the proteasome inhibitor MG132 (1 μM) and the proteasome activator IU1 (75 μM). The latter is a selective inhibitor of the proteasome-associated deubiquitinase Usp14, which limits degradation by trimming ubiquitin chains from substrates (Lee et al., 2010). Its inhibition by IU1 thus accelerates proteasome degradation of ubiquitin-tagged proteins. Throughout time, MG132 led to increased UiFC intensity, whilst IU1 decreased it in comparison to the DMSO-treated condition (Figures 1D,E), which is in accordance with their opposing effects on proteasome activity and concomitant effect on the levels of ubiquitinated proteins. Moreover, we used an inhibitor of deubiquitinases with broad specificity, PR619 (at 1 μM; Altun et al., 2011), which inhibits removal of Ub chains, thus leading to overall enhanced cellular polyubiquitination (Pinto et al., 2016). Indeed, a gradual increase in UiFC signal was observed when neurons were exposed to PR619 (Figures 1D,E). Therefore, we demonstrated that UiFC is sensitive to changing levels of endogenous ubiquitination in neurons.

**Figure 2:**
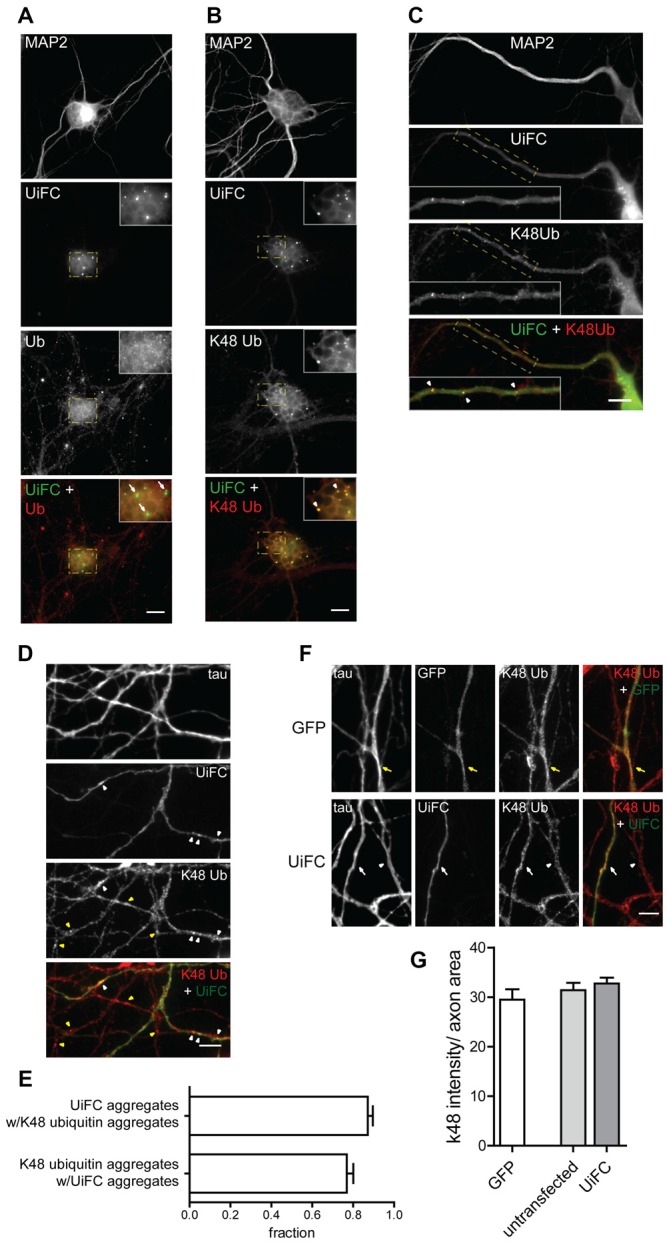
**Colocalization of UiFC with K48 ubiquitin chains in neurons. (A–D)** Pattern of UiFC expression and distribution in hippocampal neurons. Expression of UiFC in neurons was followed by immunostaining for **(A)** ubiquitin (red) or **(B–D)** K48-linkage specific ubiquitin (red). MAP2 and tau were used as dendritic and axonal marker respectively. UiFC has a diffuse pattern in neurons with distinct UiFC aggregates (white arrowheads) on **(A,B)** somas, **(C)** dendrites and **(D)** axons, which perfectly colocalize with **(B–D)** K48 ubiquitin staining, **(A)** but not ubiquitin. **(A–C)** Enlarged image on inset corresponds to dashed boxes. Scale bars are 10 μm. **(D)** Yellow arrowheads correspond to K48 ubiquitin aggregates in untransfected axons. **(E)** Fraction of UiFC aggregates colocalizing with K48 ubiquitin aggregates and vice-versa. **(F)** Assessment of UiFC effect on the levels of K48 ubiquitination along axons. Expression of GFP or UiFC was followed by staining for tau and K48 polyubiquitin (red). K48 ubiquitin staining does not vary between axons expressing GFP (yellow arrow), UiFC (white arrow) and untransfected neighbors (arrowhead). **(D,F)** Scale bars are 5 μm. **(G)** Intensity of K48 ubiquitin per axon area. Statistical significance by Kruskal-Wallis test followed by the Dunn’s multiple comparison test. A total of 39 (GFP), 90 (untransfected) and 55 (UiFC) axonal segments analyzed from three independent experiments.

We next assessed the distribution pattern of UiFC in neurons. To accomplish so, UiFC-expressing samples were immunostained for ubiquitin or for K48-linked ubiquitin (Newton et al., 2008) along with the somatodendritic marker MAP2 (Figures 2A,B). UiFC showed a diffused staining in somas with occasional bright aggregates (Figures 2A,B), which had also been previously observed decorating HeLa cells (Chen et al., 2013). Close attention to dendrites (Figure 2C) and axons (Figure 2D) revealed similar pattern of UiFC distribution. Remarkably, both diffuse and aggregated UiFC signal colocalized with staining for K48-specific ubiquitin chains (Figures 2B–D), but not with ubiquitin staining (Figure 2A). In axons, the great majority of UiFC aggregates (87%) were also positive for aggregates of K48 ubiquitin (Figure 2E) and vice-versa (77%), thus suggesting that UiFC aggregates faithfully represent endogenous sites of enrichment of K48 ubiquitinated conjugates along axons. This is in agreement with previous biochemical and imaging analysis showing that UiFC preferentially detects K48 ubiquitin chains over K11 or K63, most likely due to favorable chain conformation allowing for Venus reconstitution (Chen et al., 2013).

**Figure 3:**
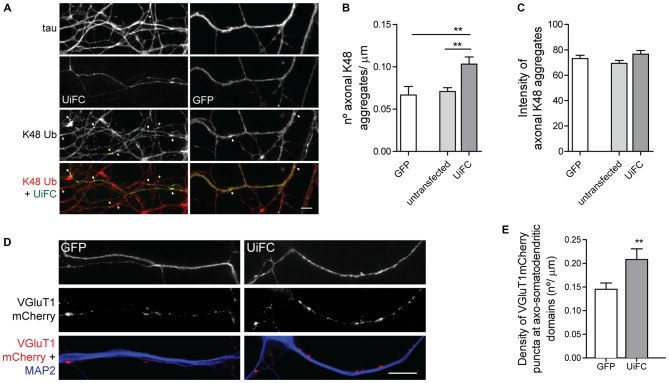
**UiFC expression enhances presynaptic clustering in axo-somatodendritic domains. (A)** Assessment of UiFC effect on the density and intensity of axonal K48 aggregates. Neurons expressing either GFP or UiFC were stained for tau and K48 ubiquitin (red). UiFC expression increases the density of K48 aggregates. White and yellow arrowheads indicate K48 ubiquitin aggregates in transfected and untransfected axons, respectively. Scale bar represents 5 μm. **(B,C)** Quantitative values of **(B)** number of K48 aggregates per axonal length and **(C)** mean intensity of axonal K48 aggregates. Statistical significance by Kruskal-Wallis test followed by the Dunn’s multiple comparison test (**p < 0.01 between indicated conditions). A total of 39 (GFP), 90 (untransfected) and 55 (UiFC) axonal segments analyzed from three independent experiments. **(D)** Effect of UiFC on presynaptic assembly onto somatodendritic elements. Neurons expressing UiFC or a control GFP plasmid together with the presynaptic reporter VGluT1mCherry were stained for MAP2 (blue), GFP and mCherry (red). UiFC-expressing axons have higher density of somatodendritic VGluT1 puncta. Scale bar represents 10 μm. **(E)** Averaged density of VGluT1mCherry puncta at sites of axo-somatodendritic contact expressed in number of puncta per axonal length. Statistical significance by Mann Whitney test (**p < 0.01). Data from 40 (GFP) and 44 (UiFC) axonal segments from two independent experiments.

Binding of UiFC’s ubiquitin interacting motifs to growing ubiquitinated chains on substrates may interfere with the normal stability of the protein or its dynamic ubiquitination. An *in vitro* ubiquitination reaction with increasing doses of UiFC fragments, showed that UiFC does not interfere with normal polyubiquitination (Chen et al., 2013). To assess whether neuronal expression of UiFC alters levels of its targets in axons, neurons expressing UiFC or a control GFP-expressing plasmid were stained for K48 polyubiquitin (Figure 2F). No differences in the total intensity of K48 ubiquitin signal were observed between axons expressing GFP, UiFC or untransfected neighbors (Figures 2F,G), thereby demonstrating that under basal conditions UiFC does not affect stability of K48 ubiquitin-tagged conjugates in axons. We then assessed whether UiFC alters the distribution pattern of K48 ubiquitin in axons by quantifying the density and intensity of K48 ubiquitin aggregates. Although no differences were observed in the intensity of K48 ubiquitin aggregates (Figures 3A,C), UiFC-expressing axons have a higher number of aggregates in comparison to untransfected neighbors and GFP-expressing ones (Figures 3A,B). These results reveal that UiFC alters the pattern of K48 ubiquitination along axons with more K48 ubiquitin aggregates populating the axon. This effect is likely due to enhanced aggregation of polyubiquitinated conjugates through interaction with UiFC’s ubiquitin interacting motifs, generating highly stable macromolecular complexes.

We then asked whether such enhanced aggregation of K48 ubiquitin by UiFC has a functional role in the axon or merely represents a functionless artifact. We have previously proposed that localized accumulation of proteasome-related polyubiquitinated conjugates triggers presynaptic assembly (Pinto et al., 2016). Considering that UiFC enhances the number of aggregates of K48 ubiquitin along axons (Figures 3A,B), we asked whether this would be positively reflected on the density of presynaptic clusters being formed onto a postsynaptic partner. To investigate this possibility, we co-expressed UiFC or control GFP with the excitatory presynaptic reporter VGluT1mCherry, which consists of a fusion version of the vesicular glutamate transporter 1 (VGluT1) to the fluorescent protein mCherry. Cultures were then stained for MAP2 to locate dendrites. In order to compensate for UiFC and VGluT1mCherry fluorescence loss following the staining procedure, cultures were also stained for GFP (the antibody used also recognizes Venus) and mCherry to enhance the signal of both reporters. Attention was given to axons expressing both VGluT1mCherry and UiFC or GFP at sites of contact with MAP2^+^ structures. Higher density of VGluT1mCherry clusters at axo-somatodendritic regions was observed in axons expressing UiFC (Figures 3D,E). The fact that UiFC enhances K48 ubiquitin aggregation on axons and also increases the number of presynaptic terminals argues in favor of the role of K48 ubiquitination in presynaptic assembly proposed in our recent study (Pinto et al., 2016). Moreover, it clearly indicates that UiFC enhancing effect on the aggregation pattern of axonal K48 ubiquitin can exert a biological signaling role.

In this set of results, we demonstrate that UiFC signal can be used in neurons for tracking and monitoring K48 polyubiquitination. We further show that aggregates of K48 ubiquitinated conjugates can be found in axons and reliably detected with UiFC signal. It should be noted, however, that UiFC expression produces an enhancing effect on K48 aggregation along axons.

### Stable Axonal Aggregates of K48 Polyubiquitin

**Figure 4:**
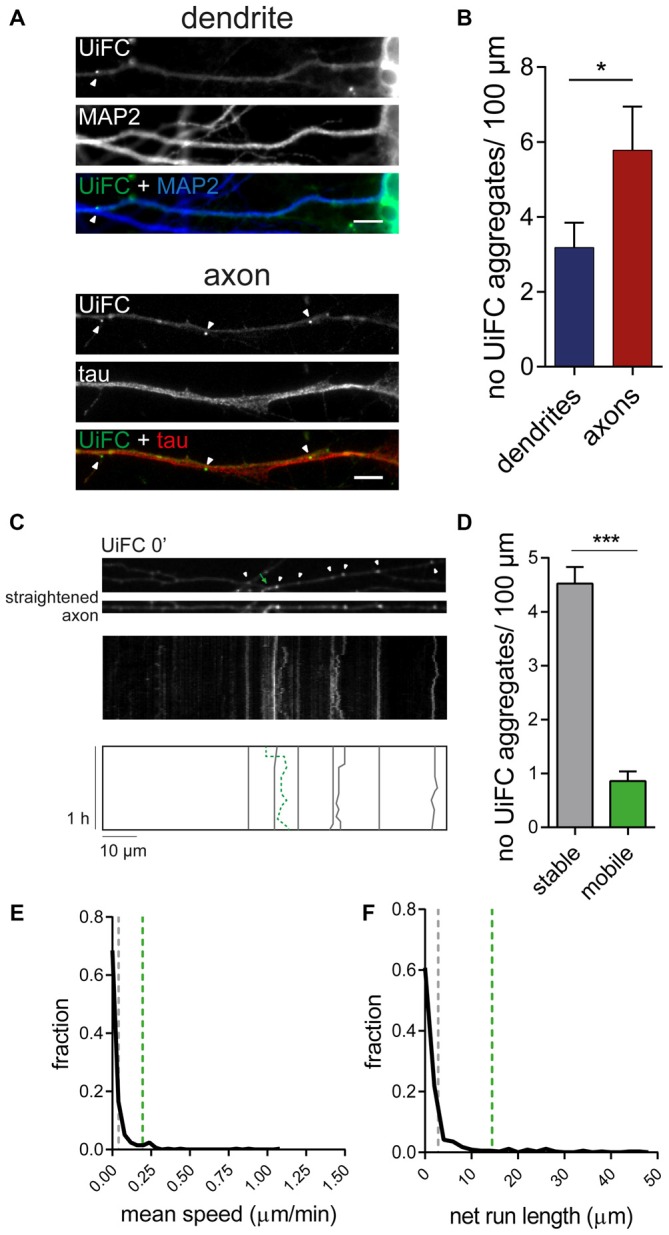
**Dynamics of UiFC aggregates along axons. (A)** Distribution of UiFC aggregates in dendrites and axons. Immunostaining for MAP2 (blue) and tau (red) was performed to identify dendrites (top) and axons (bottom) of neurons expressing UiFC (green). Arrowheads indicate UiFC aggregates. Scale bars represent 5 μm. **(B)** Quantification of number of UiFC aggregates per 100 μm of dendritic and axonal segment shows a higher density of UiFC aggregates in axons than in dendrites. Results are represented as Mean ± SEM. Statistical significance assessed by Mann Whitney test (*p < 0.05). A total of 35 dendritic and 25 axonal segments were analyzed from three independent experiments. **(C)** Mobility of UiFC aggregates along axons. Time-lapse imaging, every 1 min for 1 h, was performed in UiFC-expressing axons to evaluate aggregates’ mobility. Top, Representative segment of a UiFC-expressing axon at the beginning of the time lapse. Arrowheads and arrow correspond to stable and mobile UiFC aggregates, respectively. Middle, representative kymograph obtained from a continuous movie at 1 min/frame (1 h total time) showing trajectories of UiFC aggregates. Bottom, schematic representation of UiFC trajectories. Gray lines and dashed green line represent tracks of UiFC aggregates at arrowheads and arrow, respectively. UiFC aggregates along axons are mostly stable. **(D)** Quantification of the number of mobile [mean instant speed greater than 0.05 μm/min and net run length greater than twice aggregates’ width (0.08 μm)] and stationary UiFC aggregates per 100 μm of axon length. Results are shown as Mean ± SEM. Statistical significance assessed by Wilcoxon paired *t*-test (***p < 0.001). A total of 93 axonal segments were analyzed from three independent experiments. **(E,F)** Fraction distribution of individual UiFC aggregates’ **(E)** mean instant speed (μm/min) and **(F)** net run length (μm). Dashed gray and green lines indicate averaged mean for stable and mobile UiFC aggregates, respectively. Data from 334 individual UiFC aggregates (285 stable and 49 mobile) from three independent experiments.

We have recently shown that enhanced presynaptic concentration of proteins in an ubiquitinated state functions as a trigger for presynaptic assembly. In particular, K11 and K48 polyubiquitin chains (Pinto et al., 2016), which normally drive proteins to proteasome clearance (Sadowski et al., 2012). Hence, deep characterization of the distribution and dynamics of accumulated K48 ubiquitination along axons is of immediate relevance. We therefore directed our attention to the UiFC aggregates dispersedly observed in neurons (Figure 2). Quantification of the density of UiFC aggregates showed that on average approximately three and six aggregates can be found per 100 μm of dendritic and axonal length, respectively (Figures 4A,B). The difference in the density of UiFC aggregates between dendrites and axons (Figures 4A,B) likely reflects different subcellular needs and involvement in distinct events.

We then sought to examine the properties of axonal UiFC aggregates. Considering that the axon is densely populated with mobile packets of material actively transported along the axon (Maday et al., 2014), we first questioned about the mobility rate of UiFC aggregates. We were mostly interested in understanding whether UiFC aggregates represented stable platforms of polyubiquitinated conjugates or mobile ubiquitin-tagged material. To accomplish so, we imaged UiFC-positive axons every 1 min for a total time of 1 h and converted signal along axons into kymographs for analysis of UiFC aggregates’ movement (Figure 4C). Tracks for each UiFC aggregate were traced (Figure 4C, bottom) and their moving behavior analyzed. Most of the aggregates were relatively stable in approximately the same position, showing only minor dislocations along the axon length throughout the imaging period (Figure 4C, white arrowheads and correspondent gray lines). In order to have a quantitative idea of the general moving behavior of axonal UiFC aggregates, we divided them into two groups: stable and mobile aggregates. For a UiFC aggregate to be considered as stable two requirements had to be met: display speed values lower than 0.05 μm/min [below the rate of the slower component of slow axonal transport (Maday et al., 2014)]; and dislocate less than the length of twice their width. Most of UiFC aggregates found along the axon were stable (approximately 5/100 μm of axon; Figures 4C,D). In contrast, less than one UiFC aggregate per 100 μm displayed considerable movement within a 1 h interval (Figures 4C,D, green arrow and correspondent dashed green line). Indeed, for the majority of UiFC aggregates, their mean speed and net displacement were close to zero (Figures 4E,F), thus further reinforcing that the majority were stably maintained in the same axonal site.

**Figure 5:**
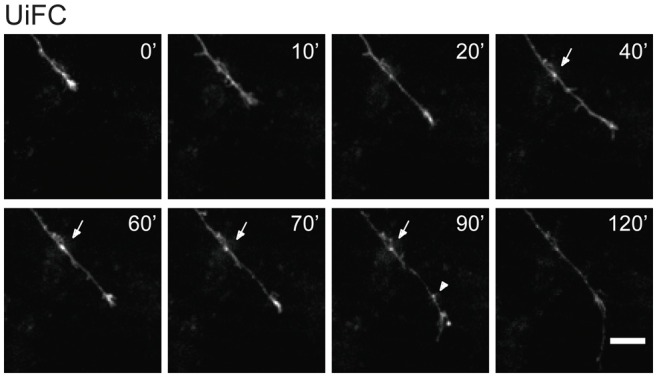
**New UiFC aggregates are formed in a navigating axon.** Hippocampal neurons cultured for 7 days were transfected with UiFC and imaged every 10 min for a minimum of 2 h with a special focus on navigating growth cones. As an axon grows, persistent (arrow) or transient (arrowhead) UiFC aggregates are formed. Scale bar is 10 μm.

In few occasions, kymographs revealed new aggregates being formed and others lost from view along the axonal shaft. Interestingly, as an axon grows and extends through the culture, dynamic formation of new UiFC aggregates was observed (Figure 5). Whilst some persisted only for a relatively short period of time (Figure 5, arrowhead), others steadily remained in the same spot of its first appearance (Figure 5, arrow). Therefore, aggregates of K48 polyubiquitin with limited mobility populate axons and are formed as an axon navigates.

### Enrichment of K48 Polyubiquitination at Presynaptic Sites

**Figure 6:**
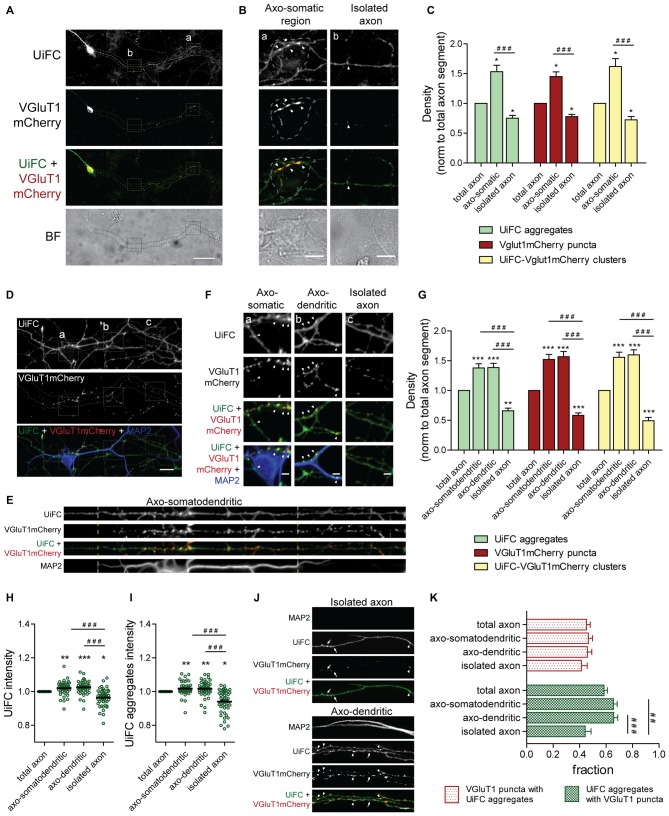
**Distribution of UiFC aggregates along axons is biased towards axonal regions contacting somas and dendrites and presynaptic sites. (A)** Differential distribution of axonal UiFC aggregates and presynaptic clusters between somatic and non-somatic axonal domains. Dual expression of UiFC (green) and the presynaptic reporter VGluT1mCherry (red) was performed in hippocampal neurons. Along axons, UiFC aggregates and VGluT1mCherry puncta were preferentially located at soma contacting axonal regions. Dashed area encircles axon of interest expressing UiFC and VGluT1mCherry. Scale bar represents 50 μm. **(B)** Enlarged images of boxes in **(A)** showing axonal segments contacting somas (a, axo-somatic) and without contact (b, isolated axon). Dashed lines outline somatic regions detected in the brightfield. Higher density of both UiFC aggregates and VGluT1mCherry puncta were observed at somas. Arrowheads indicate UiFC-VGluT1mCherry clusters. Scale bars represent 10 μm. **(C)** Averaged density of UiFC aggregates (green), VGluT1mCherry puncta (red) and UiFC-VGluT1mCherry clusters (yellow) per axonal length at segments contacting somas and isolated ones. Results are normalized to density along the total length of each axon. UiFC-VGluT1mCherry clusters correspond to colocalization events between UiFC aggregates and VGluT1mCherry puncta. Twenty axonal segments (length between 50 and 300 μm) were analyzed from three independent experiments. **(D)** Distribution of axonal UiFC aggregates and presynaptic clusters at somatodendritic structures and isolated axons. Following dual expression of UiFC (green) and VGluT1mCherry (red), staining for MAP2 (blue), GFP and mCherry was performed. Higher density of axonal UiFC aggregates and VGluT1mCherry puncta at MAP2^+^ structures. Dashed area encircles axon of interest. Scale bar represents 20 μm. **(E)** Straightened images of dashed area in **(D)**. Dashed lines highlight sites of somatodendritic contact. **(F)** Enlarged images of boxes in **(D)** showing axonal segments contacting somas (a), dendrites (b) and isolated axons (c). Arrowheads indicate UiFC-VGluT1mCherry clusters. Scale bars represent 5 μm. **(G)** Averaged density of UiFC aggregates (green), VGluT1mCherry puncta (red) and UiFC-VGluT1mCherry clusters (yellow) per axonal length at axonal segments contacting somatodendritic and dendritic elements and isolated ones. Results are normalized to density along the total length of each axon. **(H,I)** Intensity of **(H)** UiFC and **(I)** UiFC aggregates along the axon at sites with and without contact with MAP2^+^ structures. For each axonal segment, results were normalized to intensity values in the total axon. **(J)** UiFC aggregates co-localize with presynaptic clusters. Colocalization between UiFC (green) and VGluT1mCherry (red) signal along axons showed that the majority of UiFC aggregates overlap with presynaptic puncta. Arrowheads and arrows point to clusters of UiFC-VGluT1mCherry and UiFC aggregates alone, respectively. Scale bar represents 5 μm. **(K)** Fraction of axonal UiFC aggregates colocalizing with VGluT1mCherry puncta (green bars) and vice-versa (red bars) at sites with and without somato and/or dendritic contact. **(C,G–I,K)** Results are shown as Mean ± SEM. Statistical significance by Kruskal-Wallis test followed by the Dunn’s multiple comparison test (*p < 0.05, **p < 0.01 and ***p < 0.001 when compared to total axon and ^##^*p* < 0.01 and ^###^*p* < 0.001 between indicated conditions). **(G–I,K)** 45 axonal segments (length between 50 and 400 μm) were analyzed from three independent experiments.

In order to better understand the biological significance of K48 ubiquitin accumulations, we studied the distribution of UiFC aggregates along axons. We asked whether their density would be influenced upon synapse formation. In other words, are UiFC aggregates preferentially located at the presynaptic domain of axo-somatic synapses? To answer this question, we fixed cells following UiFC expression and compared the density of UiFC aggregates along axonal segments with or without contact with neuronal somas (Figures 6A,B). As it can be observed, along the length of the same axon, UiFC aggregates are found at higher density at axonal domains that represent sites of axo-somatic synapses rather than “bare” segments (Figures 6A–C, green bars).

The fact that UiFC aggregates are preferentially placed at soma-contacting axonal domains raises the question of whether they are at presynaptic sites or in their vicinity. To investigate this possibility, we co-expressed UiFC with the presynaptic reporter VGluT1mCherry. VGluT1mCherry puncta were mainly found at soma-contacting axonal domains (Figures 6A–C, red bars), thus demonstrating that somas induced in axons formation of presynaptic clusters. Extra-somatic VGluT1mCherry puncta present in isolated axons (Figures 6B,D, red bars) are likely to represent mobile packets of synaptic vesicles that later give rise to terminals (Kraszewski et al., 1995; Ahmari et al., 2000). Colocalization analysis between UiFC aggregates and VGluT1mCherry puncta demonstrated that the majority of axonal UiFC aggregates colocalized perfectly (white arrows) with VGluT1mCherry puncta (Figures 6J,K, green bars). Moreover, clusters in which a VGluT1mCherry puncta and a UiFC aggregate colocalize are preferentially found at axonal domains in contact with somas, axo-somatic regions, as opposed to isolated extra-somatic regions (Figures 6B,C, yellow bars). These results show that enrichment of K48 polyubiquitin along axons is more likely at soma-triggered sites of presynaptic clustering.

We then asked whether such preferential distribution of K48 ubiquitin enriched aggregates is also observed at axonal sites in contact with dendrites. As previously described (Figure 3D), cultures expressing UiFC and VGluT1mCherry were stained for MAP2, GFP and mCherry (Figure 6D). In accordance to the concentration of UiFC aggregates at axo-somatic regions (Figures 6A–C), higher density of axonal UiFC aggregates is observed at regions juxtaposing MAP2^+^ dendrites (axo-dendritic regions) in comparison to isolated axonal segments (Figures 6D–G, green bars). In a way of reinforcing the synaptic nature of these domains, a higher density of VGluT1mCherry puncta was also observed (Figures 6D–G, red bars). We then determined the density of clusters containing both UiFC and VGluT1mCherry and again observed their higher concentration on axo-dendritic domains (Figures 6D–G, yellow bars). It should be noted that density of axonal K48 ubiquitinated aggregates and presynaptic puncta at MAP2^+^ somatodendritic regions (Figures 6D–G) is similar to the one obtained at somatic domains detected in brightfield (Figures 6A–C). Moreover, no changes were observed between axo-somatodendritic and axo-dendritic domains (Figures 6D–G), thus revealing that distribution of UiFC aggregates and VGluT1mCherry puncta have no clear preference for dendrites over somas.

To guarantee that increased detection of UiFC aggregates at somatodendritic domains is due to a real increase in the number of aggregates rather than global enhanced intensity of the reporter, total UiFC signal along the axon was quantified (Figure 6H). UiFC intensity is higher at domains juxtaposed to somatodendritic structures in comparison to isolated segments (Figure 6H). We then asked whether such difference arises from a higher intensity of UiFC aggregates at these regions. Indeed, axonal aggregates of UiFC are more intense when in contact with somas and dendrites (Figure 6I). Therefore, somatodendritic contact elicits in the contacting axon appearance of UiFC aggregates of higher intensity.

In order to clarify the extent to which UiFC aggregates and VGluT1mCherry puncta overlap, we quantified the fraction of VGluT1 puncta at UiFC aggregates, and vice-versa, along an axon at sites of contact and outside (Figures 6J,K). Approximately 45% of VGluT1 puncta colocalized with UiFC aggregates regardless of contact with somas and dendrites (Figures 6J,K). On the contrary, UiFC aggregates are more prone to overlap with presynaptic puncta at sites of somatodendritic contact (approximately 65% against 44% along an isolated axon; Figures 6J,K). These results suggest that the localization of an axonal UiFC aggregate at somatodendritic contact sites increases the likelihood of a presynaptic bouton being formed. Although at a much lower density (Figures 6C,G) and fraction of isolated UiFC aggregates (Figures 6J,K), UiFC-VGluT1mCherry clusters can be found in an isolated axonal segment. This may be indicative of periods of co-transport or formation of orphan terminals. On the other hand, the fact that a fraction of axo-somatodendritic VGluT1mCherry puncta do not colocalize with UiFC aggregates (Figures 6J,K) suggests one of two possibilities: (1) their presence does not coincide with sites of polyubiquitin enrichment or (2) concentration of polyubiquitin occurred at an earlier time-point.

Altogether, our data indicate that the majority of aggregates enriched in K48 polyubiquitin are present at presynaptic sites of axo-somatodendritic synapses.

### Enhanced K48 Ubiquitination at Sites of Presynaptic Formation

**Figure 7:**
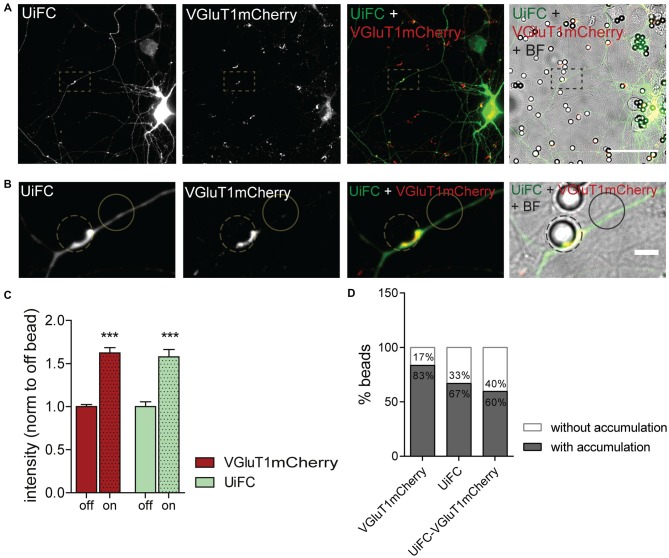
**Enhanced UiFC fluorescence at newly formed presynaptic terminals. (A)** UiFC signal on bead-induced presynaptic clusters. Neurons were co-transfected with UiFC (green) and VGluT1mCherry (red) and poly-D-lysine (PDL)-coated beads added for a total of 8 h. UiFC signal was greatly enhanced in bead-induced presynaptic clusters. Scale bar represents 50 μm. **(B)** Enlarged images of box in **(A)**. Scale bar represents 5 μm. **(C)** Quantitative values of VGluT1mCherry and UiFC intensity at on- and off-bead sites (all beads included in the analysis). Results are normalized to off-bead and shown as mean ± SEM. Statistical analysis by Wilcoxon paired *t*-test (***p < 0.001 between on and off for each marker). **(D)** Percentage of beads inducing accumulation of UiFC, VGluT1mCherry and both on contacting axons in relation to adjacent off-sites. A total of 152 beads were analyzed from two independent experiments.

We next asked whether K48 ubiquitination concentrates at the site of nascent presynaptic clusters. Beads with a cationic-coating have been shown to constitute a spatiotemporally controlled way of triggering formation of functional presynaptic sites (Burry, 1980, 1982; Burry et al., 1986; Lucido et al., 2009). Upon their contact with neuronal cells, appearance of presynaptic-like elements occurs as rapidly as after 1–2 h of contact (Burry, 1982; Lucido et al., 2009; Suarez et al., 2013). Beads induce aggregation of presynaptic material in axonal contact sites as well as cytoskeleton rearrangements in the form of enhanced localized actin filaments (Lucido et al., 2009), which have a prominent role in the initial formation of the presynapse (Zhang and Benson, 2001; Lucido et al., 2009; Nelson et al., 2013). Likewise, considering that enhanced UPS-related ubiquitination propels presynaptic differentiation (Pinto et al., 2016), we hypothesized that K48 polyubiquitin signals become enriched at bead-contacting axonal sites. To address this hypothesis, expression of UiFC and VGluT1mCherry in neurons was followed by addition of PDL-coated beads and incubation for 8 h (Figures 7A,B). Intense clustering of VGluT1mCherry was observed at bead sites in comparison to an off-bead adjacent domain (Figures 7A–C), in accordance to previous studies (Lucido et al., 2009). More importantly, UiFC intensity was increased in these axonal-bead contact sites (Figures 7A–C), thus indicating a strong accumulation of K48 ubiquitination at sites of induction of presynaptic formation. In order to determine the fraction of responsive beads, we calculated the percentage of beads inducing enhanced accumulation of VGluT1mCherry, UiFC and both in contacting axons (Figure 7D). The great majority of beads triggered recruitment of the presynaptic reporter (83%), the K48 polyubiquitination reporter (67%) and both (60%; Figure 7D). Importantly, from the pool of beads accumulating UiFC the majority also led to VGluT1mCherry clustering (89%), thus suggesting that localized accumulation of K48 polyubiquitination strongly relates to presynaptic clustering.

**Figure 8:**
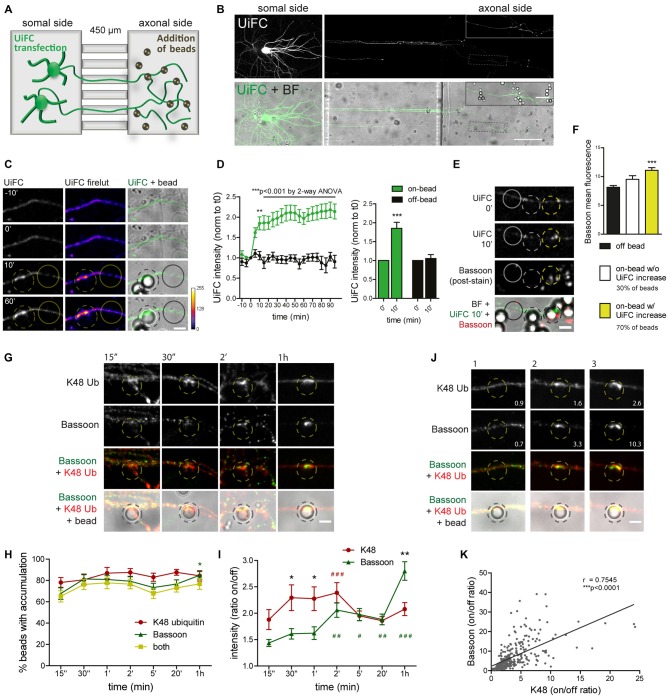
**Rapid increase in UiFC intensity on beads correlates with presynaptic formation. (A)** Schematic representation and **(B)** representative image of a microfluidic platform to monitor changes in axonal polyubiquitination upon bead contact. **(A,B)** Expression of UiFC at the somal side of microfluidic devices resulted in UiFC^+^ axons reaching the axonal compartment, to which beads were subsequently added and their effect on the contacting axon monitored by live-cell imaging. Thicker branches on soma side represent dendrites that do not cross into the axonal compartment. **(B)** UiFC signal was differently adjusted between right and left images to prevent over-saturation in somas. Enlarged image on right upper corner corresponds to dashed box. Scale bars represent 20 μm and 100 μm. **(C)** Profile of axonal UiFC intensity upon bead contact at on- and off-bead sites. Individual frames of a time-lapse series showing the initial contact of a bead (dashed circle) with a UiFC-expressing axon (firelut, green). Images were taken every 5 min. Bead contact resulted in a rapid and strong increase in local UiFC intensity, as opposed to an off-bead adjacent site (solid circle). **(D)** Left, Quantification of UiFC intensity at beads (on-bead, green) and adjacent sites (off-bead, black) throughout time. Statistical significance was assessed by 2-way ANOVA (***p < 0.001 and **p < 0.01 between on- and off-bead at each time-point). Right, Comparison of UiFC intensity values at 0 min and 10 min post-bead contact for both on- and off-bead sites. Statistical analysis by Wilcoxon paired *t*-test (***p < 0.001 when compared to the correspondent 0 min time-point). Results are normalized to 0 min, which corresponds to the frame preceding addition of beads, and shown as Mean ± SEM. A total of 193 beads and equivalent off-bead sites analyzed from two independent experiments. **(E)** Correlation between enhanced UiFC signal on beads and posterior presynaptic clustering. Retrospective labeling for the active zone marker Bassoon (red) was performed 4–5 h post-UiFC (green) time-lapse on beads. Clustering of presynaptic material was greater on beads that displayed increased UiFC intensity shortly after bead contact (yellow vs. white dashed circle). Solid circle indicates off-bead site. **(F)** Averaged raw Bassoon intensity values at off- and on-bead sites. Beads were divided into two pools according to their effect on axonal UiFC intensity at 10 min contact: beads not changing UiFC signal (~30%, white bar) and beads eliciting increases in UiFC signal above off-bead levels (~70%, yellow bar). Statistical significance by Kruskal-Wallis test followed by the Dunn’s multiple comparison test (***p < 0.001 compared to off-bead). Data from 191 beads (57 and 134 beads without and with UiFC increase at 10 min bead contact) and equivalent off-bead sites from two independent experiments. **(G)** Time-course of K48 ubiquitin and Bassoon accumulation on beads. Axons in microfluidic devices were exposed to beads for the indicated periods of time and stained for Bassoon (green) and K48 ubiquitin (red). Both K48 ubiquitin and Bassoon rapidly build up on beads. Initial strong accumulation of K48 ubiquitin is followed by Bassoon clustering. The scale bar is 5 μm. **(H)** Percentage of beads with accumulation of K48 ubiquitin and Bassoon. **(I)** Ratio of K48 ubiquitin and Bassoon intensity between bead and corresponding adjacent site. **(H,I)** For individual K48 ubiquitin (red) and Bassoon (green) timelines, statistical significance by Kruskal-Wallis test followed by the Dunn’s multiple comparison test (^###^*p* < 0.001, ^##^*p* < 0.01 and ^#^*p* < 0.05 compared to 15’). Comparison between K48 ubiquitin and Bassoon at each time-point by 2-way ANOVA (*p < 0.05). Data from 112 (15”), 76 (30”), 89 (1’), 134 (2’), 154 (5’), 129 (20’) and 114 (1 h) beads from 3–4 independent experiments. **(J)** Correlation between the amount of K48 ubiquitin and Bassoon at beads (2 h contact). Values at bottom right are the ratios of intensities at on- and off-site for each bead shown. The scale bar is 5 μm. **(K)** Positive correlation between K48 ubiquitin and Bassoon at beads (r, Spearman’s rank correlation coefficient; ***p < 0.001). A total of 297 beads analyzed from two independent experiments.

In order to look specifically to the response of developing axons not exposed to influences from somatodendritic elements or glia, we used a microfluidic system for the compartmentalization of axons (Taylor et al., 2005; Pinto et al., 2016). In microfluidic devices, two facing compartments are connected by a set of microgrooves, which are sufficiently long and narrow to prevent crossing of somas and dendrites while allowing axons to reach a physically and fluidically isolated chamber (Figure 8A; Taylor et al., 2005). This system has been fulfilling the rising demand for technical approaches capable of overcoming the neuronal polarity issue, and in fact it has been used as a tool for the study of axon-intrinsic mechanisms (Hengst et al., 2009; Taylor et al., 2009; Magnifico et al., 2013; Cristovão et al., 2014; Neto et al., 2014; Kim and Jaffrey, 2016; Pinto et al., 2016).

We used time-lapse imaging to study the dynamics of axonal K48 ubiquitin accumulation on nascent presynaptic terminals. Expression of UiFC at the compartment in which neuronal cells have been plated results in UiFC-expressing axons crossing the microgrooves into the axonal side (Figures 8A,B). We therefore used this system to monitor axon-autonomous effects on K48 polyubiquitin accumulation on beads following their addition to the axonal chamber and contact with resident axons (Figures 8A,B). Bead contact induced in isolated axons a rapid (10 min) local increase in the intensity of the polyubiquitination reporter, UiFC, as opposed to adjacent sites (Figures 8C,D). The sudden increase in UiFC signal remains equally high until the end of the time-lapse (90 min) with no further considerable increases (Figures 8C,D). Importantly, this effect is not the result of increased axonal volume on bead-contacting domains (Pinto et al., 2016). To examine the relevance of initial accumulation of K48 ubiquitin to presynaptic clustering on beads, we stained axons for the presynaptic active zone marker Bassoon 4–5 h post-imaging (Figure 8E). Beads were divided into two groups according to their positive or negative effect on UiFC signal at 10 min post-contact (approximately 70% and 30% of beads, respectively; Figure 8F). In comparison to an adjacent off-bead site, clustering of Bassoon was enhanced on beads that were capable of rapidly increasing UiFC intensity upon contact (Figures 8E,F). These results indicate that accumulation of K48 polyubiquitinated conjugates at the initial stages of presynaptic formation is related to subsequent formation of the active zone.

Assembly of presynaptic terminals has been proposed to respect a temporal order of material recruitment (Friedman et al., 2000; Suarez et al., 2013). In order to rigorously examine the timing of K48 ubiquitin enrichment in relation to recruitment of presynaptic material to the nascent terminal, axons in microfluidic devices were stained for K48 ubiquitin and Bassoon at different times post-bead contact (Figures 8G–I). Bassoon is trafficked in Piccolo-Bassoon transport vesicles (Zhai et al., 2001; Shapira et al., 2003), mobile packets of active zone material which are believe to be first-comers to the presynapse (Friedman et al., 2000; Suarez et al., 2013), thereby making it an excellent temporal marker of presynaptic assembly. Initial clustering of Bassoon on beads occurred surprisingly fast, with detectable increases only after 15 s of contact (ratio between on- and off-bead of approximately 1.4 in 67% of beads), followed by progressive clustering until 1 h (2.8 on/off ratio in 85% of beads; Figures 8G–I). Enrichment of K48 ubiquitin on beads was also detected at the shortest time-interval tested (1.9 on/off ratio in 78% of beads; Figures 8G–I). However, contrary to Bassoon, with K48 an initial period of intense enrichment was rapidly observed (approximately 2.3 on/off ratio between 30 s to 2 min) followed by a slight decrease at around 5 min (Figures 8G–I), which is comparable to the level of UiFC change on beads at these later time-points (Figures 8C,D). So, K48 ubiquitin and Bassoon are hastily accumulated on beads in a synchronized manner. An early intense accumulation of K48 ubiquitin is followed by sustained Bassoon clustering. We and others (Lucido et al., 2009; Suarez et al., 2013; Pinto et al., 2016) have previously proposed that clustering of synaptic vesicle markers on beads occurs at a later time than the initial recruitment time for Bassoon herein observed (Figures 8G–I), which is in agreement with the proposed timeline for presynaptic assembly onto an axo-dendritic contact (Bassoon—active zone formation—accumulation of synaptic vesicles; Friedman et al., 2000). Therefore, local enrichment of K48 ubiquitin occurs coincidently to the initial recruitment of active zone material (Figures 8G–I), and precedes clustering of synaptic vesicles. Likewise, enhanced accumulation of a proteasome reporter on beads was observed prior to clustering of a synaptic vesicle marker (Pinto et al., 2016).

Finally, we tested for a correlation between the amount of accumulated K48 ubiquitin on beads and Bassoon clustering. Do beads with higher levels of K48 ubiquitin tend to cluster more presynaptic material and vice-versa? A positive correlation was found between K48 ubiquitin and Bassoon on beads at 2 h contact (Figures 8J,K). We can therefore conclude that beads’ efficiency to cluster Bassoon is positively correlated to the amount of accumulated K48 polyubiquitin signals. Altogether, this set of results reveals that K48 ubiquitinated conjugates accumulate locally at the initial stages of presynaptic assembly and is intimately associated to efficient clustering of active zone material.

### Presynaptic Clustering Requires Axonal E1 Ubiquitin-Activating Enzyme Activity

**Figure 9:**
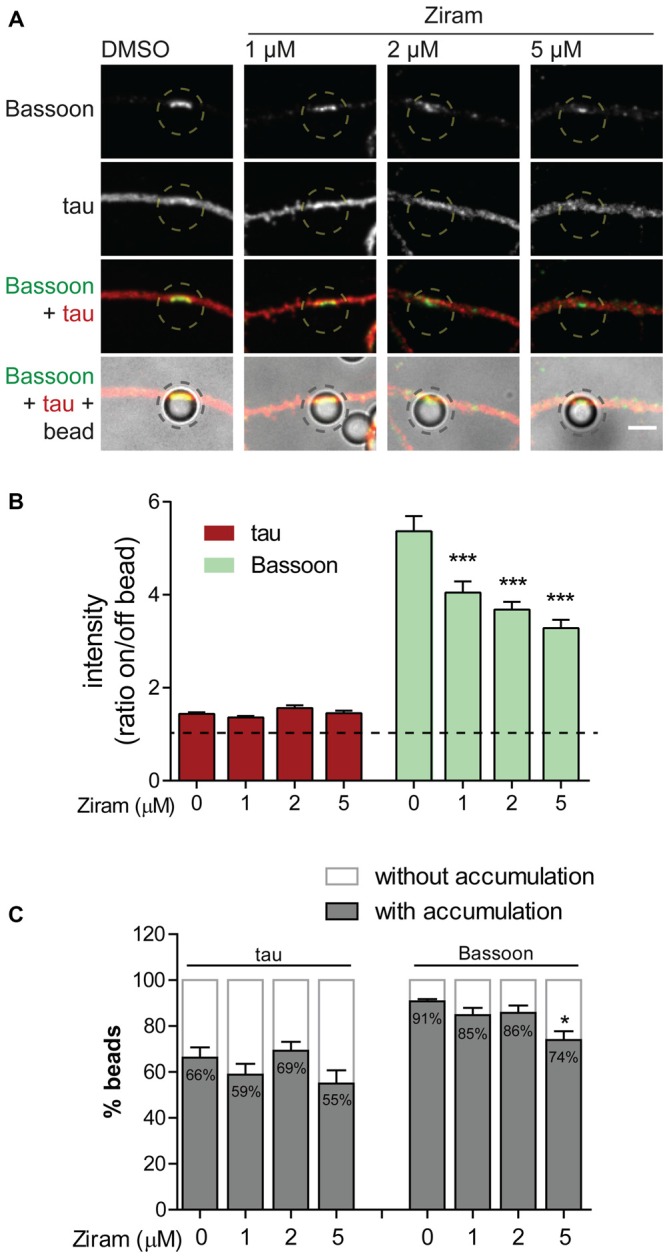
**Presynaptic clustering on beads requires axonal E1-ubiquitin activation. (A)** Requirement of protein ubiquitination for presynaptic clustering on beads. Incubation of beads for 2 h in the axonal side of microfluidic devices was performed in the presence or absence of the E1 ubiquitin activating enzyme inhibitor ziram (1, 2 and 5 μM) followed by immunostaining for tau (red) and Bassoon (green). Inhibition of axonal E1-mediated ubiquitination reduced clustering of presynaptic material on beads. Scale bar represents 5 μm. **(B)** Ratio of tau and Bassoon intensity between bead and corresponding adjacent site. Dashed line indicates 1. **(C)** Percentage of beads inducing accumulation of bassoon and tau on contacting axons in relation to adjacent off-sites. **(B,C)** Statistical significance by Kruskal-Wallis test followed by the Dunn’s multiple comparison test (***p < 0.001 and *p < 0.05 compared to 0 μM). A total of 525, 535, 421 and 414 beads (0, 1, 2 and 5 μM ziram, respectively) analyzed from five independent experiments.

To confirm the biological relevance of ubiquitination as a functional means to presynaptic formation, we investigated dependence of presynaptic clustering on E1-mediated ubiquitination. The enzymatic cascade catalyzing ubiquitination of substrates comprises sequential activity of the E1 ubiquitin-activating enzyme, E2 ubiquitin-conjugating enzyme and E3 ubiquitin-ligase (Neutzner and Neutzner, 2012; Heride et al., 2014). In order to prevent ubiquitination we used ziram, an inhibitor of the E1 ubiquitin-activating enzyme. This inhibitor reduces E1 activity by preventing formation of E1-ubiquitin conjugates (Chou et al., 2008; Rinetti and Schweizer, 2010), thus compromising subsequent transfer of ubiquitin moieties to the E2 active site and consequently ubiquitination. The axonal chamber of microfluidic devices was treated with increasing doses of ziram (0, 1, 2 and 5 μM) in the presence of beads. A pre-incubation of 10 min with ziram was performed to guarantee that E1 was inhibited prior initial axon contact with beads. Importantly, because ziram loses efficiency in the reduction of E1-ubiquitin conjugation in longer incubation periods (Rinetti and Schweizer, 2010), we decided to shorten the experiment time to 2 h, after which devices were fixed and stained for the axonal marker tau and Bassoon (Figure 9A). In control conditions (vehicle), beads elicited intense clustering of Bassoon in the juxtaposed axonal segment in relation to adjacent regions, with a high ratio between Bassoon intensity at on- and off-bead (Figures 9A,B). Increasing doses of ziram progressively decreased the amount of Bassoon clustered on beads, whilst having no effect on tau (Figures 9A,B), an axonal microtubule-associated protein that diffusely distributes along healthy axons (Kosik and Finch, 1987; Black et al., 1996). The lack of change on tau distribution between on- and off-bead sites indicates that ziram effect is specific towards clustering of presynaptic components. The fact that no substantial changes were observed in the percentage of beads inducing Bassoon clustering above off-bead levels (Figure 9C), shows us that ziram, rather than diminishing the number of beads capable of triggering clustering, affects the amount of recruited presynaptic material per bead. Hence, clustering of presynaptic material on beads requires activity of the E1-ubiquitin activating enzyme at the axon level, thus emphasizing the crucial role of local ubiquitination in the mechanisms governing presynaptic differentiation.

## Discussion

**Figure 10:**
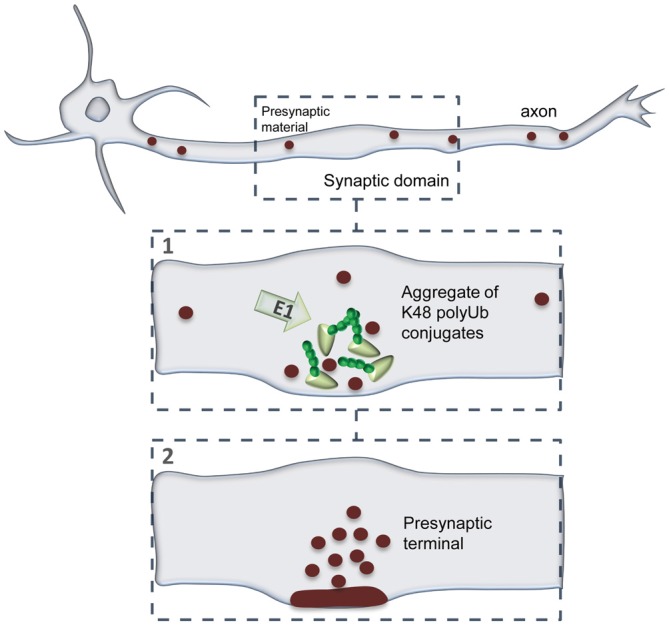
**A model of presynaptic formation at sites of enhanced K48 polyubiquitination.** At axonal domains contacting a synaptic partner, strong accumulation of K48 ubiquitin at nascent sites occurs at the early moments of recruitment of active zone material (Bassoon; 1). Then, formation of a functional presynaptic terminal will be achieved by sustained recruitment of active zone material, as well as synaptic vesicles, which will be properly clustered and assembled (2). Presynaptic formation is dependent on the activity of axonal E1-mediated ubiquitination.

In this study, we evaluated the dynamics of K48 ubiquitination along axons and its correlation to sites of presynaptic formation. We observed that the axon contains aggregates of K48 polyubiquitinated conjugates whose majority is relatively stable (Figures 2–5). Secondly, these aggregates are mainly found at sites of clusters of presynaptic material and in the axonal counterpart of axo-somatodendritic synapses (Figure 6). We then questioned about enrichment of K48 polyubiquitination at the site of nascent presynaptic terminals from a temporal perspective and found that it occurs simultaneously to the initial recruitment of Bassoon (Figures 7, 8). An early temporal window of intense K48 ubiquitin enrichment is followed by progressive active zone clustering (Figure 8). Finally, we attributed a fundamental role for axonal ubiquitination, with a particular focus on the local activity of E1 ubiquitin-activating enzyme, on presynaptic assembly (Figure 9). The data reported here together with our previous findings (Pinto et al., 2016), suggests that site-specific enrichment of K48 polyubiquitinated conjugates requires axonal ubiquitination and acts as an initial local signal for presynaptic differentiation (Figure 10). However, the precise molecular mechanism underlying this phenomenon has yet to be determined.

Throughout this study we made use of the recently developed UiFC approach to monitor K48 ubiquitination along axons. UiFC is capable of sensing changing levels of ubiquitination (Figure 1), does not alter stability of K48 ubiquitinated targets and shows high degree of colocalization with endogenous K48 ubiquitin in neurons (Figure 2). Our results also show that UiFC mildly enhanced the density of axonal K48 ubiquitin aggregates (Figure 3). Nevertheless, UiFC robustly identifies sites of enhanced accumulation of K48 ubiquitin along axons, and so, can be used to monitor dynamics of K48 ubiquitination. Such conclusion is based on the following observations: (1) ~87% of axonal UiFC aggregates colocalize with endogenous aggregates of K48 ubiquitin, thus showing that UiFC aggregates are *bona fide* sites of K48 ubiquitin enrichment in axons; (2) K48 aggregates can be found in untransfected axons and not only on UiFC-expressing ones, thus indicating that under basal conditions axonal sites of accumulated K48 ubiquitination exist (Figure 2D). UiFC further enhances/stabilizes their presence in axons (Figures 3A,B); (3) UiFC expression leads to higher density of presynaptic clusters onto somatodendritic structures (Figures 3D,E), which suggests that UiFC-enhanced aggregation of K48 ubiquitin in axons has a functional role in the axon; and (4) distribution of UiFC aggregates in axons is not random but biased towards contact sites with somatodendritic elements and presynaptic clusters (Figure 6).

Axonal sites of enriched K48 polyubiquitination are in close association to presynaptic sites. Our analysis of UiFC aggregates along axons of hippocampal neurons shows that approximately 85% of K48 polyubiquitinated aggregates are stationary, barely moving from the same spot, whilst 15% are mobile, although with a low movement speed (Figure 4). Of important note is the fact that the average instant speed by which these mobile aggregates move (0.2 μm/min) does not conform to that of fast axonal transport or slow axonal transport of cytosolic proteins (14–280 μm/min and 0.7–7 μm/min, respectively; Maday et al., 2014; Roy, 2014), thus discarding the possibility that K48 polyubiquitinated aggregates are actively transported by microtubule-mediated axonal transport. Instead, it is tempting to speculate that they constitute freely-diffusible aggregates, similar to a previously described diffusion-like motion of the proteasome along axons (Otero et al., 2014). As for the stationary aggregates, their confined location suggests that they represent stable hot-spots enriched with K48 polyubiquitinated conjugates in axons. The fact that new aggregates are stably formed as an axon extends through its environment (Figure 5); that they are mainly present in axonal domains in contact with somas and dendrites and that the majority colocalize with clusters of presynaptic vesicles (Figure 6), gives support to the idea that differentiation of presynaptic sites in a developing axon is assisted by enriched sites of K48 polyubiquitin-tagged conjugates. It is important, however, to stress the possibility that UiFC aggregates correspond to axonal protein aggregates where K48-tagged proteins await to be degraded. Indeed, one may surmise a scenario in which clearance of ubiquitinated aggregates either by proteasome degradation or intense deubiquitination follows their signaling role in the developing axon.

Localized accumulation of K48 polyubiquitinated conjugates and dependence on axonal ubiquitination underlie presynaptic clustering. Reconstitution of UiFC fluorescence and abundance of endogenous K48 ubiquitin were greatly and rapidly potentiated upon axon contact with synapse formation-inducing beads (Figure 8). Importantly, this local phenomenon occurred simultaneously to the initial clustering of active zone material (Bassoon) on beads. An early period of intense enrichment of K48 ubiquitin is followed by sustained recruitment of active zone material (Figure 8), thus suggesting a sequential ordering of events leading to presynaptic assembly that comprises enhanced on-site ubiquitination as an initial step. The temporal dynamics of accumulation of K48 polyubiquitin conjugates on beads, assessed by UIFC, correlates remarkably with the local decrease in proteasome activity that we previously observed at bead-contacting axons (from 10 min of bead contact on; Pinto et al., 2016). Likewise, the same proportion of beads (approximately 70%) triggers in overlapping axons a decreased rate of degradation of a proteasome reporter (Pinto et al., 2016) as well as higher UiFC levels (Figure 8). Together, these data open the likely possibility that accumulation of K48 ubiquitin signals on beads is the direct outcome of a localized halt in UPS degradation. It should be noted, however, that staining for the endogenous pool of K48 ubiquitin at shorter time intervals (in the seconds range) reveals faster enrichment (Figures 8G–I), which would not probably be possible to observe with UiFC due to the time required for reconstitution of Venus fluorescence (Chen et al., 2013). In addition, clustering of presynaptic material on beads (Figure 9), as well as axonal proteasome inhibition-induced presynaptic assembly (Pinto et al., 2016), requires axonal E1-catalyzed ubiquitin activation. These results are in agreement with previous work in *C. elegans* (DiAntonio et al., 2001) and mouse lines (Burgess et al., 2004; Chen et al., 2011; Bachiller et al., 2015) that assign paramount importance to protein ubiquitination for presynaptic formation, however not further explored.

Noticeably, the effect of beads on the local levels of K48 ubiquitination and the dependence of presynaptic clustering on ubiquitin conjugation have also been observed when considering actin polymerization (Lucido et al., 2009). Indeed, presynaptic clustering on beads depends on localized reorganization of actin filaments (Lucido et al., 2009). In addition, nascent presynapses along axons are associated with enhanced levels of filamentous actin (Zhang and Benson, 2002) and increased K48 polyubiquitin signal (Pinto et al., 2016). In terms of actin cytoskeleton, it is currently believed that formation of a filamentous actin network recruits synaptic vesicles to discrete sites along the axon by acting as a scaffold for nascent presynapses (Nelson et al., 2013). It is therefore possible that actin and ubiquitin function together to erect a local platform for the recruitment of presynaptic material. Thus, it is essential to unveil how multiple intra-axonal events may interact synergistically and cooperatively to yield presynaptic differentiation efficiently.

Appendage of ubiquitin chains is a versatile way of functionally altering proteins and thereby controlling diverse cellular processes. An interesting finding tell us that only 5% of total ubiquitin in the brain can be found as polyubiquitin chains on substrates (Kaiser et al., 2011). This raises the possibility that cells are equipped to respond rapidly to new intracellular polyubiquitination signals, rather than requiring proteins to remain accumulated in their polyubiquitinated state for long periods. In the same line of thought, together with our recent work (Pinto et al., 2016), we herein propose that quick and site-specific enrichment of K48 polyubiquitinated conjugates occurs in the early steps of presynaptic assembly to assist in clustering of material. This model suggests that K48-linked ubiquitin chains are able to exert a different biological function, other than signaling proteasomal removal. It further suggests that the same type of ubiquitin chain attached to a protein may engage it into diverse roles depending on signal duration, localization and available downstream ubiquitin interpreters and effectors. For instance, β-catenin, an intervenient of the Wnt signaling pathway that among other functions induces presynaptic clustering (Hall et al., 2000), can undergo enhanced stability (Hay-Koren et al., 2011) or be targeted for degradation (Dimitrova et al., 2010) both by attachment of K11 polyubiquitin chains. Nonetheless, the means by which locally enriched K48 polyubiquitin signals trigger presynaptic formation remains unclear. One possibility is that an ubiquitinated enriched platform, composed of either axonal cytoskeleton proteins or presynaptic scaffolding elements, created at axonal domains undergoing synaptogenesis recruits presynaptic material. Indeed, several proteins involved in synaptogenesis and structural elements of the presynaptic terminal can be found in an ubiquitinated state (Franco et al., 2011; Na et al., 2012). Therefore, identification of the ubiquitinated conjugates featuring presynaptogenic properties would be a key issue to disclose, so that the mechanism could be better understood. Ubiquitin proteomics to material clustered on beads at different time-points after initial contact would help to characterize the presynaptic ubiquitome on demand for presynaptic formation.

Neurodevelopmental diseases may arise from abnormal function of the UPS. It is suggested that genetic mutations in enzymes of the ubiquitination cascade or abnormal expression of ubiquitin signaling machinery may underlie neurodevelopmental defects (Hegde and Upadhya, 2011). For instance, mutations in the E1 ubiquitin-activating enzyme or its reduced levels with subsequent disruption of ubiquitin homeostasis contribute to the development of spinal muscular atrophy (Ramser et al., 2008; Wishart et al., 2014). Furthermore, the E3 ubiquitin-ligase UBE3A is implicated in both Angelman syndrome (Kishino et al., 1997; Matsuura et al., 1997) and autism spectrum disorders (Baron et al., 2006; Glessner et al., 2009). In schizophrenia, ubiquitin signaling is hindered due to reduced expression of several involved proteins, with concomitant decreases in monomeric and conjugated ubiquitin (Middleton et al., 2002; Altar et al., 2005; Rubio et al., 2013). This substantial prevalence as a causative factor for the onset of neurodevelopmental diseases emphasizes the need to fully understand and characterize the physiological role of ubiquitin and the proteasome. Particularly, given the data presented here, it is of great importance to pursue the role of localized ubiquitination for presynaptic differentiation.

## Author Contributions

Study conception and design by MJP and RDA. MJP: conducted the experiments. RDA and MJP: wrote the article. JRP and ROC: performed experiments for revision.

## Funding

This work was supported by the individual grants SFRH/BD/51196/2010 (MJP), SFRH/BD/77789/2011 (JRP), SFRH/BPD/84593/2012 (ROC) by Fundação para a Ciência e Tecnologia (FCT); by FEDER through Programa Operacional Factores de Competividade—COMPETE; by national funds through FCT: PTDC/SAU-NEU/104100/2008, EXPL/NEU-NMC/0541/2012 and UID/NEU/04539/2013; and by Marie Curie Actions—International reintegration Grant, 7th Framework programme, EU.

## Conflict of Interest Statement

The authors declare that the research was conducted in the absence of any commercial or financial relationships that could be construed as a potential conflict of interest.

## Abbreviations

K: lysine
PDL: poly-D-lysine
UiFC: ubiquitination-induced fluorescence complementation
UPS: ubiquitin-proteasome system
VGluT1: vesicular glutamate transporter 1.

## References

[B1] Ahmari S. E. Buchanan J. Smith S. J. (2000). Assembly of presynaptic active zones from cytoplasmic transport packets. Nat. Neurosci. 3, 445–451. 10.1038/74814 10769383

[B2] Almeida R. D. Manadas B. J. Melo C. V. Gomes J. R. Mendes C. S. Grãos M. M.. (2005). Neuroprotection by BDNF against glutamate-induced apoptotic cell death is mediated by ERK and PI3-kinase pathways. Cell Death Differ. 12, 1329–1343. 10.1038/sj.cdd.4401662 15905876

[B3] Altar C. A. Jurata L. W. Charles V. Lemire A. Liu P. Bukhman Y.. (2005). Deficient hippocampal neuron expression of proteasome, ubiquitin and mitochondrial genes in multiple schizophrenia cohorts. Biol. Psychiatry 58, 85–96. 10.1016/j.biopsych.2005.03.031 16038679

[B4] Altun M. Kramer H. B. Willems L. I. McDermott J. L. Leach C. A. Goldenberg S. J.. (2011). Activity-based chemical proteomics accelerates inhibitor development for deubiquitylating enzymes. Chem. Biol. 18, 1401–1412. 10.1016/j.chembiol.2011.08.018 22118674

[B5] Bachiller S. Rybkina T. Porras-García E. Pérez-Villegas E. Tabares L. Armengol J. A.. (2015). The HERC1 E3 ubiquitin ligase is essential for normal development and for neurotransmission at the mouse neuromuscular junction. Cell. Mol. Life Sci. 72, 2961–2971. 10.1007/s00018-015-1878-2 25746226 PMC11113414

[B6] Baeza J. L. de la Torre B. G. Santiveri C. M. Almeida R. D. García-López M. T. Gerona-Navarro G.. (2012). Cyclic amino acid linkers stabilizing key loops of brain derived neurotrophic factor. Bioorg. Med. Chem. Lett. 22, 444–448. 10.1016/j.bmcl.2011.10.107 22119467

[B7] Baptista F. I. Pinto M. J. Elvas F. Almeida R. D. Ambrósio A. F. (2013). Diabetes alters KIF1A and KIF5B motor proteins in the hippocampus. PLoS One 8:e65515. 10.1371/journal.pone.0065515 23776493 PMC3680435

[B8] Baptista M. S. Melo C. V. Armelão M. Herrmann D. Pimentel D. O. Leal G.. (2010). Role of the proteasome in excitotoxicity-induced cleavage of glutamic acid decarboxylase in cultured hippocampal neurons. PLoS One 5:e10139. 10.1371/journal.pone.0010139 20405034 PMC2853570

[B9] Baron C. A. Tepper C. G. Liu S. Y. Davis R. R. Wang N. J. Schanen N. C.. (2006). Genomic and functional profiling of duplicated chromosome 15 cell lines reveal regulatory alterations in UBE3A-associated ubiquitin-proteasome pathway processes. Hum. Mol. Genet. 15, 853–869. 10.1093/hmg/ddl004 16446308

[B10] Black M. M. Slaughter T. Moshiach S. Obrocka M. Fischer I. (1996). Tau is enriched on dynamic microtubules in the distal region of growing axons. J. Neurosci. 16, 3601–3619. 8642405 10.1523/JNEUROSCI.16-11-03601.1996 PMC6578833

[B11] Bresler T. Shapira M. Boeckers T. Dresbach T. Futter M. Garner C. C.. (2004). Postsynaptic density assembly is fundamentally different from presynaptic active zone assembly. J. Neurosci. 24, 1507–1520. 10.1523/JNEUROSCI.3819-03.2004 14960624 PMC6730341

[B12] Burgess R. W. Peterson K. A. Johnson M. J. Roix J. J. Welsh I. C. O’Brien T. P. (2004). Evidence for a conserved function in synapse formation reveals Phr1 as a candidate gene for respiratory failure in newborn mice. Mol. Cell. Biol. 24, 1096–1105. 10.1128/mcb.24.3.1096-1105.2004 14729956 PMC321423

[B13] Burry R. W. (1980). Formation of apparent presynaptic elements in response to poly-basic compounds. Brain Res. 184, 85–98. 10.1016/0006-8993(80)90588-0 7357426

[B14] Burry R. W. (1982). Development of apparent presynaptic elements formed in response to polylysine coated surfaces. Brain Res. 247, 1–16. 10.1016/0006-8993(82)91022-8 7127105

[B15] Burry R. W. Raymond H. H. Matthew W. D. (1986). Presynaptic elements formed on polylysine-coated beads contain synaptic vesicle antigens. J. Neurocytol. 15, 409–419. 10.1007/bf01611725 3091776

[B16] Bury L. A. D. Sabo S. L. (2010). How it’s made: the synapse. Mol. Interv. 10, 282–292. 10.1124/mi.10.5.5 21045242

[B17] Chen P. C. Bhattacharyya B. J. Hanna J. Minkel H. Wilson J. A. Finley D.. (2011). Ubiquitin homeostasis is critical for synaptic development and function. J. Neurosci. 31, 17505–17513. 10.1523/JNEUROSCI.2922-11.2011 22131412 PMC3253363

[B18] Chen P. C. Qin L. N. Li X. M. Walters B. J. Wilson J. A. Wilson S. M. (2009). The proteasome-associated deubiquitinating enzyme Usp14 is essential for the maintenance of synaptic ubiquitin levels and the development of neuromuscular junctions. J. Neurosci. 29, 10909–10919. 10.1523/JNEUROSCI.2635-09.2009 19726649 PMC2766780

[B19] Chen Z. Zhong Y. Wang Y. Xu S. Liu Z. Baskakov I. V.. (2013). Ubiquitination-induced fluorescence complementation (UiFC) for detection of K48 ubiquitin chains *in vitro* and in live cells. PLoS One 8:e73482. 10.1371/journal.pone.0073482 24039955 PMC3764048

[B20] Chou A. P. Maidment N. Klintenberg R. Casida J. E. Li S. Fitzmaurice A. G.. (2008). Ziram causes dopaminergic cell damage by inhibiting E1 ligase of the proteasome. J. Biol. Chem. 283, 34696–37703. 10.1074/jbc.M802210200 18818210 PMC2596383

[B21] Collins C. A. Wairkar Y. P. Johnson S. L. DiAntonio A. (2006). Highwire restrains synaptic growth by attenuating a MAP kinase signal. Neuron 51, 57–69. 10.1016/j.neuron.2006.05.026 16815332

[B22] Cristovão G. Pinto M. J. Cunha R. A. Almeida R. D. Gomes C. A. (2014). Activation of microglia bolsters synapse formation. Front. Cell. Neurosci. 8:153. 10.3389/fncel.2014.00153 24917790 PMC4040490

[B23] Das U. Wang L. Ganguly A. Saikia J. M. Wagner S. L. Koo E. H.. (2015). Visualizing APP and BACE-1 approximation in neurons yields insight into the amyloidogenic pathway. Nat. Neurosci. 19, 55–64. 10.1038/nn.4188 26642089 PMC4782935

[B24] DiAntonio A. Haghighi A. P. Portman S. L. Lee J. D. Amaranto A. M. Goodman C. S. (2001). Ubiquitination-dependent mechanisms regulate synaptic growth and function. Nature 412, 449–452. 10.1038/35086595 11473321

[B25] Dimitrova Y. N. Li J. Lee Y. T. Rios-Esteves J. Friedman D. B. Choi H. J.. (2010). Direct ubiquitination of β-catenin by Siah-1 and regulation by the exchange factor TBL1. J. Biol. Chem. 285, 13507–13516. 10.1074/jbc.M109.049411 20181957 PMC2859511

[B26] Fang D. Kerppola T. K. (2004). Ubiquitin-mediated fluorescence complementation reveals that Jun ubiquitinated by Itch/AIP4 is localized to lysosomes. Proc. Natl. Acad. Sci. U S A 101, 14782–14787. 10.1073/pnas.0404445101 15469925 PMC522008

[B27] Feinberg E. H. VanHoven M. K. Bendesky A. Wang G. Fetter R. D. Shen K.. (2008). GFP reconstitution across synaptic partners (GRASP) defines cell contacts and synapses in living nervous systems. Neuron 57, 353–363. 10.1016/j.neuron.2007.11.030 18255029

[B28] Franco M. Seyfried N. T. Brand A. H. Peng J. Mayor U. (2011). A novel strategy to isolate ubiquitin conjugates reveals wide role for ubiquitination during neural development. Mol. Cell. Proteomics 10:M110.002188. 10.1074/mcp.M110.002188 20861518 PMC3098581

[B29] Friedman H. V. Bresler T. Garner C. C. Ziv N. E. (2000). Assembly of new individual excitatory synapses: time course and temporal order of synaptic molecule recruitment. Neuron 27, 57–69. 10.1016/s0896-6273(00)00009-x 10939331

[B30] Glessner J. T. Wang K. Cai G. Korvatska O. Kim C. E. Wood S.. (2009). Autism genome-wide copy number variation reveals ubiquitin and neuronal genes. Nature 459, 569–573. 10.1038/nature07953 19404257 PMC2925224

[B31] Hall A. C. Lucas F. R. Salinas P. C. (2000). Axonal remodeling and synaptic differentiation in the cerebellum is regulated by WNT-7a signaling. Cell 100, 525–535. 10.1016/s0092-8674(00)80689-3 10721990

[B32] Hallengren J. Chen P. C. Wilson S. M. (2013). Neuronal ubiquitin homeostasis. Cell Biochem. Biophys. 67, 67–73. 10.1007/s12013-013-9634-4 23686613 PMC3758786

[B33] Hay-Koren A. Caspi M. Zilberberg A. Rosin-Arbesfeld R. (2011). The EDD E3 ubiquitin ligase ubiquitinates and up-regulates β-catenin. Mol. Biol. Cell 22, 399–411. 10.1091/mbc.E10-05-0440 21118991 PMC3031469

[B34] Hegde A. N. Upadhya S. C. (2011). Role of ubiquitin-proteasome-mediated proteolysis in nervous system disease. Biochim. Biophys. Acta 1809, 128–140. 10.1016/j.bbagrm.2010.07.006 20674814 PMC2995838

[B35] Hengst U. Deglincerti A. Kim H. J. Jeon N. L. Jaffrey S. R. (2009). Axonal elongation triggered by stimulus-induced local translation of a polarity complex protein. Nat. Cell Biol. 11, 1024–1030. 10.1038/ncb1916 19620967 PMC2724225

[B36] Heride C. Urbé S. Clague M. J. (2014). Ubiquitin code assembly and disassembly. Curr. Biol. 24, R215–R220. 10.1016/j.cub.2014.02.002 24650902

[B37] Herzog E. Nadrigny F. Silm K. Biesemann C. Helling I. Bersot T.. (2011). *In vivo* imaging of intersynaptic vesicle exchange using VGLUT1 Venus knock-in mice. J. Neurosci. 31, 15544–15559. 10.1523/JNEUROSCI.2073-11.2011 22031900 PMC6703545

[B38] Jin Y. Garner C. C. (2008). Molecular mechanisms of presynaptic differentiation. Annu. Rev. Cell Dev. Biol. 24, 237–262. 10.1146/annurev.cellbio.23.090506.123417 18588488

[B39] Johnson-Venkatesh E. M. Umemori H. (2010). Secreted factors as synaptic organizers. Eur. J. Neurosci. 32, 181–190. 10.1111/j.1460-9568.2010.07338.x 20646052 PMC4127169

[B40] Kaiser S. E. Riley B. E. Shaler T. A. Trevino R. S. Becker C. H. Schulman H.. (2011). Protein standard absolute quantification (PSAQ) method for the measurement of cellular ubiquitin pools. Nat. Methods 8, 691–696. 10.1038/nmeth.1649 21743460 PMC3196335

[B41] Kerppola T. K. (2006). Visualization of molecular interactions by fluorescence complementation. Nat. Rev. Mol. Cell Biol. 7, 449–456. 10.1038/nrm1929 16625152 PMC2512262

[B42] Kim J. Jaffrey S. (2016). Separating neuronal compartments gives clues as to local effect of ubiquitin conjugates in synaptogenesis. J. Cell Biol. 28, 751–753. 10.1083/jcb.201603028 27022088 PMC4810311

[B43] Kishino T. Lalande M. Wagstaff J. (1997). UBE3A/E6-AP mutations cause Angelman syndrome. Nat. Genet. 15, 70–73. 10.1038/ng0197-70 8988171

[B44] Kleiger G. Mayor T. (2014). Perilous journey: a tour of the ubiquitin-proteasome system. Trends Cell Biol. 24, 352–359. 10.1016/j.tcb.2013.12.003 24457024 PMC4037451

[B45] Komander D. Rape M. (2012). The ubiquitin code. Annu. Rev. Biochem. 81, 203–229. 10.1146/annurev-biochem-060310-170328 22524316

[B46] Kosik K. S. Finch E. A. (1987). MAP2 and tau segregate into dendritic and axonal domains after the elaboration of morphologically distinct neurites: an immunocytochemical study of cultured rat cerebrum. J. Neurosci. 7, 3142–3153. 2444675 10.1523/JNEUROSCI.07-10-03142.1987 PMC6569170

[B47] Kraszewski K. Mundigl O. Daniell L. Verderio C. Matteoli M. De Camilli P. (1995). Synaptic vesicle dynamics in living cultured hippocampal neurons visualized with CY3-conjugated antibodies directed against the lumenal domain of synaptotagmin. J. Neurosci. 15, 4328–4342. 7540672 10.1523/JNEUROSCI.15-06-04328.1995 PMC6577731

[B48] Krueger S. R. Kolar A. Fitzsimonds R. M. (2003). The presynaptic release apparatus is functional in the absence of dendritic contact and highly mobile within isolated axons. Neuron 40, 945–957. 10.1016/s0896-6273(03)00729-3 14659093

[B49] Kulathu Y. Komander D. (2012). Atypical ubiquitylation–the unexplored world of polyubiquitin beyond Lys48 and Lys63 linkages. Nat. Rev. Mol. Cell Biol. 13, 508–523. 10.1038/nrm3394 22820888

[B50] Lee B. H. Lee M. J. Park S. Oh D. C. Elsasser S. Chen P. C.. (2010). Enhancement of proteasome activity by a small-molecule inhibitor of USP14. Nature 467, 179–184. 10.1038/nature09299 20829789 PMC2939003

[B51] Liao E. H. Hung W. Abrams B. Zhen M. (2004). An SCF-like ubiquitin ligase complex that controls presynaptic differentiation. Nature 430, 345–350. 10.1038/nature02647 15208641

[B52] Lucido A. L. Suarez Sanchez F. Thostrup P. Kwiatkowski A. V. Leal-Ortiz S. Gopalakrishnan G.. (2009). Rapid assembly of functional presynaptic boutons triggered by adhesive contacts. J. Neurosci. 29, 12449–12466. 10.1523/JNEUROSCI.1381-09.2009 19812321 PMC3849729

[B53] Macpherson L. J. Zaharieva E. E. Kearney P. J. Alpert M. H. Lin T. Y. Turan Z.. (2015). Dynamic labelling of neural connections in multiple colours by trans-synaptic fluorescence complementation. Nat. Commun. 6:10024. 10.1038/ncomms10024 26635273 PMC4686661

[B54] Maday S. Twelvetrees A. E. Moughamian A. J. Holzbaur E. L. F. (2014). Axonal transport: cargo-specific mechanisms of motility and regulation. Neuron 84, 292–309. 10.1016/j.neuron.2014.10.019 25374356 PMC4269290

[B55] Magnifico S. Saias L. Deleglise B. Duplus E. Kilinc D. Miquel M. C.. (2013). NAD acts on mitochondrial SirT3 to prevent axonal caspase activation and axonal degeneration. FASEB J. 27, 4712–4722. 10.1096/fj.13-229781 23975935

[B56] Matsuura T. Sutcliffe J. S. Fang P. Galjaard R. J. Jiang Y. H. Benton C. S.. (1997). De novo truncating mutations in E6-AP ubiquitin-protein ligase gene (UBE3A) in Angelman syndrome. Nat. Genet. 15, 74–77. 10.1038/ng0197-74 8988172

[B57] Middleton F. A. Mirnics K. Pierri J. N. Lewis D. A. Levitt P. (2002). Gene expression profiling reveals alterations of specific metabolic pathways in schizophrenia. J. Neurosci. 22, 2718–2729. 11923437 10.1523/JNEUROSCI.22-07-02718.2002 PMC6758309

[B58] Na C. H. Jones D. R. Yang Y. Wang X. Xu Y. Peng J. (2012). Synaptic protein ubiquitination in rat brain revealed by antibody-based ubiquitome analysis. J. Proteome Res. 11, 4722–4732. 10.1021/pr300536k 22871113 PMC3443409

[B59] Nakata K. Abrams B. Grill B. Goncharov A. Huang X. Chisholm A. D.. (2005). Regulation of a DLK-1 and p38 MAP kinase pathway by the ubiquitin ligase RPM-1 is required for presynaptic development. Cell 120, 407–420. 10.1016/j.cell.2004.12.017 15707898

[B60] Nelson J. C. Stavoe A. K. H. Colón-Ramos D. A. (2013). The actin cytoskeleton in presynaptic assembly. Cell Adh. Migr. 7, 379–387. 10.4161/cam.24803 23628914 PMC3739815

[B61] Neto E. Alves C. J. Sousa D. M. Alencastre I. S. Lourenço A. H. Leitão L.. (2014). Sensory neurons and osteoblasts: close partners in a microfluidic platform. Integr. Biol. (Camb) 6, 586–595. 10.1039/c4ib00035h 24675920

[B62] Neutzner M. Neutzner A. (2012). Enzymes of ubiquitination and deubiquitination. Essays Biochem. 52, 37–50. 10.1042/bse0520037 22708562

[B63] Newton K. Matsumoto M. L. Wertz I. E. Kirkpatrick D. S. Lill J. R. Tan J.. (2008). Ubiquitin chain editing revealed by polyubiquitin linkage-specific antibodies. Cell 134, 668–678. 10.1016/j.cell.2008.07.039 18724939

[B64] Otero M. G. Alloatti M. Cromberg L. E. Almenar-Queralt A. Encalada S. E. Pozo Devoto V. M.. (2014). Fast axonal transport of the proteasome complex depends on membrane interaction and molecular motor function. J. Cell Sci. 127, 1537–1549. 10.1242/jcs.140780 24522182

[B66] Pinto M. Almeida R. (2016). Puzzling out presynaptic differentiation. J. Neurochem. 10.1111/jnc.13702 27315450

[B65] Pinto M. J. Alves P. L. Martins L. Pedro J. R. Ryu H. R. Jeon N. L.. (2016). The proteasome controls presynaptic differentiation through modulation of an on-site pool of polyubiquitinated conjugates. J. Cell Biol. 212, 789–801. 10.1083/jcb.201509039 27022091 PMC4810304

[B67] Ramaker J. M. Swanson T. L. Copenhaver P. F. (2013). Amyloid precursor proteins interact with the heterotrimeric G protein Go in the control of neuronal migration. J. Neurosci. 33, 10165–10181. 10.1523/JNEUROSCI.1146-13.2013 23761911 PMC3682380

[B68] Ramser J. Ahearn M. E. Lenski C. Yariz K. O. Hellebrand H. von Rhein M.. (2008). Rare missense and synonymous variants in UBE1 are associated with X-linked infantile spinal muscular atrophy. Am. J. Hum. Genet. 82, 188–193. 10.1016/j.ajhg.2007.09.009 18179898 PMC2253959

[B69] Rinetti G. V. Schweizer F. E. (2010). Ubiquitination acutely regulates presynaptic neurotransmitter release in mammalian neurons. J. Neurosci. 30, 3157–3166. 10.1523/JNEUROSCI.3712-09.2010 20203175 PMC2905680

[B70] Roy S. (2014). Seeing the unseen: the hidden world of slow axonal transport. Neuroscientist 20, 71–81. 10.1177/1073858413498306 23912032 PMC3902140

[B71] Rubio M. D. Wood K. Haroutunian V. Meador-Woodruff J. H. (2013). Dysfunction of the ubiquitin proteasome and ubiquitin-like systems in schizophrenia. Neuropsychopharmacology 38, 1910–1920. 10.1038/npp.2013.84 23571678 PMC3746696

[B72] Sabo S. L. Gomes R. A. McAllister A. K. (2006). Formation of presynaptic terminals at predefined sites along axons. J. Neurosci. 26, 10813–10825. 10.1523/JNEUROSCI.2052-06.2006 17050720 PMC6674732

[B73] Sadowski M. Suryadinata R. Tan A. R. Roesley S. N. A. Sarcevic B. (2012). Protein monoubiquitination and polyubiquitination generate structural diversity to control distinct biological processes. IUBMB Life 64, 136–142. 10.1002/iub.589 22131221

[B74] Saiga T. Fukuda T. Matsumoto M. Tada H. Okano H. J. Okano H.. (2009). Fbxo45 forms a novel ubiquitin ligase complex and is required for neuronal development. Mol. Cell. Biol. 29, 3529–3543. 10.1128/mcb.00364-09 19398581 PMC2698766

[B75] Shapira M. Zhai R. G. Dresbach T. Bresler T. Torres V. I. Gundelfinger E. D.. (2003). Unitary assembly of presynaptic active zones from piccolo-bassoon transport vesicles. Neuron 38, 237–252. 10.1016/s0896-6273(03)00207-1 12718858

[B76] Siddiqui T. J. Craig A. M. (2011). Synaptic organizing complexes. Curr. Opin. Neurobiol. 21, 132–143. 10.1016/j.conb.2010.08.016 20832286 PMC3016466

[B77] Steward O. Falk P. M. (1991). Selective localization of polyribosomes beneath developing synapses: a quantitative analysis of the relationships between polyribosomes and developing synapses in the hippocampus and dentate gyrus. J. Comp. Neurol. 314, 545–557. 10.1002/cne.903140311 1814974

[B78] Suarez F. Thostrup P. Colman D. Grutter P. (2013). Dynamics of presynaptic protein recruitment induced by local presentation of artificial adhesive contacts. Dev. Neurobiol. 73, 98–106. 10.1002/dneu.22037 22648784 PMC3518747

[B79] Taylor A. M. Berchtold N. C. Perreau V. M. Tu C. H. Jeon N. L. Cotman C. W. (2009). Axonal mRNA in uninjured and regenerating cortical mammalian axons. J. Neurosci. 29, 4697–4707. 10.1523/JNEUROSCI.6130-08.2009 19369540 PMC3632375

[B80] Taylor A. M. Blurton-Jones M. Rhee S. W. Cribbs D. H. Cotman C. W. Jeon N. L. (2005). A microfluidic culture platform for CNS axonal injury, regeneration and transport. Nat. Methods 2, 599–605. 10.1038/nmeth777 16094385 PMC1558906

[B81] Unoki T. Matsuda S. Kakegawa W. Van N. T. B. Kohda K. Suzuki A.. (2012). NMDA receptor-mediated PIP5K activation to produce PI(4,5)P 2 Is essential for AMPA receptor endocytosis during LTD. Neuron 73, 135–148. 10.1016/j.neuron.2011.09.034 22243752

[B82] Wilson S. M. Bhattacharyya B. Rachel R. A. Coppola V. Tessarollo L. Householder D. B.. (2002). Synaptic defects in ataxia mice result from a mutation in Usp14, encoding a ubiquitin-specific protease. Nat. Genet. 32, 420–425. 10.1038/ng1006 12368914

[B83] Wishart T. M. Mutsaers C. A. Riessland M. Reimer M. M. Hunter G. Hannam M. L.. (2014). Dysregulation of ubiquitin homeostasis and β-catenin signaling promote spinal muscular atrophy. J. Clin. Invest. 124, 1821–1834. 10.1172/JCI71318 24590288 PMC3973095

[B84] Zhai R. G. Vardinon-Friedman H. Cases-Langhoff C. Becker B. Gundelfinger E. D. Ziv N. E.. (2001). Assembling the presynaptic active zone: a characterization of an active zone precursor vesicle. Neuron 29, 131–143. 10.1016/S0896-6273(01)00185-4 11182086

[B85] Zhang W. Benson D. L. (2001). Stages of synapse development defined by dependence on F-actin. J. Neurosci. 21, 5169–5181. 11438592 10.1523/JNEUROSCI.21-14-05169.2001 PMC6762826

[B86] Zhang W. Benson D. L. (2002). Developmentally regulated changes in cellular compartmentation and synaptic distribution of actin in hippocampal neurons. J. Neurosci. Res. 69, 427–436. 10.1002/jnr.10313 12210837

